# A combination of conserved and diverged responses underlies *Theobroma cacao*’s defense response to *Phytophthora palmivora*

**DOI:** 10.1186/s12915-024-01831-2

**Published:** 2024-02-16

**Authors:** Noah P. Winters, Eric K. Wafula, Benjamin J. Knollenberg, Tuomas Hämälä, Prakash R. Timilsena, Melanie Perryman, Dapeng Zhang, Lena L. Sheaffer, Craig A. Praul, Paula E. Ralph, Sarah Prewitt, Mariela E. Leandro-Muñoz, Diego A. Delgadillo-Duran, Naomi S. Altman, Peter Tiffin, Siela N. Maximova, Claude W. dePamphilis, James H. Marden, Mark J. Guiltinan

**Affiliations:** 1 IGDP Ecology, The Pennsylvania State University, 422 Huck Life Sciences Building, University Park, PA 16803 USA; 2 Huck Institutes of the Life Sciences, The Pennsylvania State University, University Park, PA USA; 3 Department of Biology, The Pennsylvania State University, University Park, PA USA; 4 IGDP Plant Biology, The Pennsylvania State University, University Park, PA USA; 5 Department of Plant and Microbial Biology, University of Minnesota, St. Paul, MN USA; 6 Department of Plant Science, The Pennsylvania State University, University Park, PA USA; 7 Department of Statistics, The Pennsylvania State University, University Park, PA USA; 8 CATIE, Tropical Agricultural Research and Higher Education Center, Turrialba, Costa Rica; 9 Department of Ecology and Genetics, University of Oulu, Oulu, Finland; 10 Sustainable Perennial Crops Laboratory, U.S. Department of Agriculture-Agricultural Research Service, Beltsville, MD USA; 11 Colombian Corporation for Agricultural Research (AGROSAVIA), Mosquera, Colombia

**Keywords:** Plant-defense, Evolution, Cacao, Phytophthora, RNA-seq, Genomics

## Abstract

**Background:**

Plants have complex and dynamic immune systems that have evolved to resist pathogens. Humans have worked to enhance these defenses in crops through breeding. However, many crops harbor only a fraction of the genetic diversity present in wild relatives. Increased utilization of diverse germplasm to search for desirable traits, such as disease resistance, is therefore a valuable step towards breeding crops that are adapted to both current and emerging threats. Here, we examine diversity of defense responses across four populations of the long-generation tree crop *Theobroma cacao* L., as well as four non-cacao *Theobroma* species, with the goal of identifying genetic elements essential for protection against the oomycete pathogen *Phytophthora palmivora*.

**Results:**

We began by creating a new, highly contiguous genome assembly for the *P. palmivora-*resistant genotype SCA 6 (Additional file 1: Tables S1-S5), deposited in GenBank under accessions CP139290-CP139299. We then used this high-quality assembly to combine RNA and whole-genome sequencing data to discover several genes and pathways associated with resistance. Many of these are unique, i.e., differentially regulated in only one of the four populations (diverged 40 k–900 k generations). Among the pathways shared across all populations is phenylpropanoid biosynthesis, a metabolic pathway with well-documented roles in plant defense. One gene in this pathway, caffeoyl shikimate esterase (CSE), was upregulated across all four populations following pathogen treatment, indicating its broad importance for cacao’s defense response. Further experimental evidence suggests this gene hydrolyzes caffeoyl shikimate to create caffeic acid, an antimicrobial compound and known inhibitor of *Phytophthora spp*.

**Conclusions:**

Our results indicate most expression variation associated with resistance is unique to populations. Moreover, our findings demonstrate the value of using a broad sample of evolutionarily diverged populations for revealing the genetic bases of cacao resistance to *P. palmivora*. This approach has promise for further revealing and harnessing valuable genetic resources in this and other long-generation plants.

**Supplementary Information:**

The online version contains supplementary material available at 10.1186/s12915-024-01831-2.

## Background

For thousands of years humans have worked to incorporate a wide variety of desirable traits into crops through breeding. This process of artificial selection causes genetic bottlenecks and the subsequent erosion of diversity can be detrimental to further crop improvement [1, 2], which raises the strong possibility that extant genetic variation in wild ancestors could be a rich source of agronomically valuable alleles [2–4].

Harnessing the genetic diversity of wild populations is a particularly attractive possibility for genes affecting pathogen resistance. This is because balancing (diversifying) selection often maintains genetic variation at loci that are co-evolving with locally abundant pathogens [5–8]. When populations are spread across broad geographic areas and gene flow is low, this co-evolution creates a rich spatial tapestry of alleles conferring resistance to a diverse set of microbes. Evaluating the effect that alleles, sampled broadly across populations, have on disease resistance is therefore a valuable step towards breeding crops that are adapted to both current and emerging threats. In this study, we examine whether genotypes from wild populations of the tree crop *Theobroma cacao* L. (diverged 40 k–900 k generations) can be used to efficiently identify genes conferring resistance to the oomycete pathogen *Phytophthora palmivora* [9]. Like all plants with long generation times (~5 years in *T. cacao*), this cannot be accomplished by breeding and back-crossing for mapping genes of interest, suggesting our methods may be foundational for future studies of other slow-to-mature plant species.

*Theobroma cacao* L., the seeds of which are the raw material for chocolate, is a tropical understory plant native to the Amazon basin [10–12]. Cocoa and cocoa butter, the products created by fermenting, drying, and processing cacao seeds (“beans”), form the basis of a chocolate and confectionary market worth approximately $100 billion [11, 13]. Cacao genotypes are distributed across at least thirteen strongly differentiated population groups that are hypothesized to have evolved in partial isolation created by ancient ridgelines, glacial refugia, and/or human management [11, 14–17]. Divergence times among populations are hypothesized to be between 40,000 and 900,000 generations [9]. While there is some genetic evidence for human-mediated genetic bottlenecks during and after domestication, most cacao germplasm is thought to be unaffected by domestication [14, 15]. Moreover, many widely cultivated cacao varieties, such as those cultivated in West Africa and Indonesia, are derived from a small number of accessions from the Pound collection or Trinitario hybrids [18–20].

Annual yield loss in cacao is caused by a variety of pests and pathogens, the worst of which is black pod rot [10]. Black pod rot is caused by four *Phytophthora* species and accounts for 10 to 30% of pre-harvest yield loss [13, 21]. The two most damaging members of this quartet are *P. megakarya* and its sister species *P. palmivora* [22–24]. Native to southeast Asia, *P. palmivora* is a generalist pathogen that causes extensive yield loss to a range of hosts, including cacao, oil palm, and papaya [25–27]. There are numerous efforts to increase resistance to black pod rot through breeding. However, breeding programs for tree crops like cacao are extremely difficult and time consuming, taking decades to produce commercially viable clones [28]. Moreover, small mapping populations and, until recently, low marker density make identification of quantitative trait loci (QTL) difficult, identifying large genomic regions containing hundreds or even thousands of genes [29–31].

Despite these difficulties, several breeding programs have generated high-yielding clones with partial resistance to black pod rot [28, 29, 31–34]. These programs, while successful, have been centered around a limited number of resistant genotypes collected in the 1930s. Most alleles conferring resistance to black pod rot are, therefore, derived from a small set of parents, and limited diversity leaves clones predisposed to breakthrough infections by rapidly evolving pathogens [35]. Thus, generating clones durably resistant to pathogen challenge requires consideration of the genetic diversity in cacao’s many wild populations scattered throughout the Central and South American lowland tropics [11].

Here, we test the hypothesis that wild populations represent diverse and potentially valuable sources of genetic variation by examining defense responses across four populations of *Theobroma cacao* L and four non-cacao *Theobroma* species. Through the use of genomic, transcriptomic and metabolomic data, collected in a unified experimental design, we identified both conserved and diverged components of cacao’s defense response. Our results indicate that wild populations of crop species offer far greater genetic diversity than any single individual or narrowly selected set of genotypes and can thus provide a diverse array of novel alleles for crop improvement and that studies using this approach can identify genes affecting pathogen resistance in samples representing a single generation.

## Methods

### Genotype selection and plant propagation

**Fig. 1:**
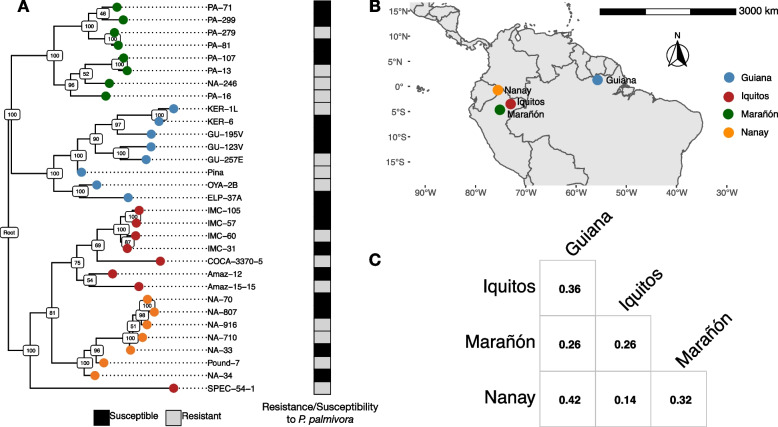
*T. cacao* population centers include genotypes that are resistant and susceptible to *P. palmivora*. **A** Maximum likelihood phylogeny of *T. cacao* genotypes based on 23,439 SNPs. White and gray boxes beside the phylogeny indicate whether genotypes were considered resistant (gray) or susceptible (black) to *P. palmivora* according to Fister et al. [36]. Numbers on the nodes indicate bootstrap support and colors at the tips indicate population membership: Guiana (blue), Iquitos (red), Marañón (green), and Nanay (orange). **B** Map displaying approximate center of current distribution for each of the four populations sampled for this study (locations are from Cornejo et al. [15]). **C** Pairwise F_ST_ estimates for each population

We selected 31 cacao genotypes for experimentation based on their resistance/susceptibility to the black pod rot causing pathogen *Phytophthora palmivora* [36]. Selected genotypes were from four populations distributed across the Amazon basin (Fig. 1). The resistant genotypes were as follows: (Guiana) Ker 1L, GU 257E, Pina, and OYA 2B; (Iquitos) IMC 60, COCA 3370/5, SPEC 54/1, and Amaz 15/15; (Marañón) NA 246, PA 13, PA 16, and PA 279; (Nanay) NA 7/10, Pound 7, and NA 916. The susceptible genotypes were: (Guiana) ELP 37A, GU 123 V, GU 195 V, and Ker 6; (Iquitos) Amaz 12, IMC 105, IMC 31, IMC 57; (Marañón) PA 107, PA 299, PA 81, and PA 71; (Nanay) NA 70, NA 807, NA 33, and NA 34. Current population centers were taken from Cornejo et al. [15]. Phylogenetic relationships among genotypes were inferred from 23,439 SNPs using SNPhylo (-l 0.6 -m 0.1 -M 0.1 -P snphylo -a 10) [37]. SNPs were obtained according to the variant calling pipeline outlined in the “Genome scan for selection” section of the “Methods”. SNPhylo further filtered these variants using a combination of linkage disequilibrium (-l 0.6), minor allele frequency (-m 0.1), and missing rate (-M 0.1). Material was imported as grafted plantlets from the International Cocoa Collection at CATIE (IC3), Costa Rica. The importation and subsequent growth of these plants were done following the requirements of and with permits from the USDA APHIS (copies available on request). From the grafted plants we created rooted cuttings according to a previously described method [38]. Single node, semi-hardwood cuttings were made from plants with an approx. 0.5 cm stem diameter. Each leaf was cut in half. We submerged the woody portion of each cutting in rooting hormone [1:1 IBA potassium salt and NAA, 0.1 g each in 50 mL 50% EtOH] and placed them in wet sand (Quikrete, medium grade), so that the leaf petiole was just above the surface. Finally, we placed the cuttings into a misting chamber (every 10 min for 6 s) surrounded by shade cloth and supplemented natural light using LED lights (16 h photoperiod, 6am–10 pm).

Once cuttings developed roots and new leaves (approx. 4 weeks after cutting), they were transplanted into D40H D-pots from Stuewe (Tangent, OR). Peat mix was used to plug the bottom of the pots before filling them with a wetted mixture of 4:2:1 Perlite:Sand:Turface. The rooted cuttings were gown with drip irrigation and watered 3 times per day: at 8 am for 10 min, at 12 pm for 6 min, and at 6 pm for 6 min. Finally, plants were incubated in the misting chamber for 2 weeks to allow them to recover before being transferred to a temperature and humidity-controlled greenhouse. Plants were then grown in a greenhouse under 80–90% relative humidity, 76 °C at night, and 83 °C during the day. Of approximately 300 cuttings, 141 developed into healthy plants that were used for further experimentation. The number of replicates per genotype, population, and resistance/susceptibility class varied (Additional file 2: Fig. S1).

### Transcriptome experimental design and treatment

To randomize environmental variation in the greenhouse (conditions described above), plants were distributed across 6 trays (~30 plants / tray) and trays were distributed across 2 adjacent benches (< 60 cm apart) (Additional file 2: Fig. S2). Trays on each bench were paired with a tray on the other bench. The plants in one tray of each pair were treated with pathogen; the plants in the other tray in the pair were treated with the control. We randomized the placement of plants in each tray, with position of the same genotype mirrored on each of the paired trays. Thus, for each pair of plants within a genotype, one would receive pathogen treatment and one would receive control treatment. If there was an odd number of plants for a given genotype, or if a genotype only had one representative plant, the odd-numbered individual was paired with an individual within the same population *and* resistance/susceptibility class [36]. If a genotype within the same population and resistance/susceptibility class was unavailable, we used a genotype in the same resistance/susceptibility class from a different population. The plants were moved to their respective positions 1 week before the experiment.

*P. palmivora* strain Gh-ER1349 was cultured on V8 media as previously described [39]. Briefly, plugs of pathogen were taken out of liquid nitrogen 3 weeks before the experiment, dried, and placed on V8 agar. Plates were placed in the dark at 27 °C. 1.5 weeks before the experiment, pathogen cultures were sub-cultured onto new V8 plates. Finally, 2 days before the experiment, *P. palmivora* plates were once again sub-cultured to create 120 thin (10 mL) V8 agar plates. Plates were then incubated in the dark at 27 °C until the day of the experiment.

Prior to inoculation, 2 leaves from each plant were selected for inoculation based on size, health, and developmental stage. All leaves were graded as stage D, D/E (transitioning from D to E) or E [40]. Inoculation was done on the abaxial side of the selected leaves using either 1.5-cm mycelia plugs taken from the growing edge of the culture, or 1.5-cm plugs of the V8 control. Inoculations were done an hour after sunset and green headlamps were worn to limit the effect of light on the plants. Six agar plugs of either pathogen mycelia or V8 control were placed on each of the selected leaves, avoiding veins or damaged portions of the leaf as much as possible. After all 6 plugs were placed, each leaf was sprayed with a fine mist of water to limit desiccation of the agar plug. After 8 h, leaves were collected following the same order as inoculation. Both leaves were carefully removed from each plant, making sure agar plugs remained attached. The leaves were then placed on a cutting board and a 1.75-cm cork borer was used to excise leaf discs with each agar plug at the center of each disc. This ensured a small amount of tissue surrounding the plugs was cut from each leaf. The agar plugs were then removed and 12 leaf discs (6 from each of 2 leaves) were pooled the into a single 2-mL tube. Tubes were immediately flash frozen in liquid nitrogen before being stored at −80 °C.

### Sample preparation and sequencing

Tissue was ground using pre-chilled (−80 °C) stainless steel beads (2 × 2.3 mm, and 1 × 3.2 mm) in a Qiagen (Hilden, Germany) TissueLyzer for 3 rounds of 40 s. Tubes were re-frozen after each round to prevent thawing. Once tissue was ground into a fine powder, samples were once again stored at − 80 °C.

RNA was extracted from 100 mg of ground tissue using a protocol adapted from Thermo Fisher Scientific’s small scale RNA isolation protocol (Publication No. MAN0000243) for PureLink™ Plant RNA Reagent (Life Technologies, Carlsbad, CA, USA). The following modifications were made: Extraction buffer (1 mL) made according to US Patent US6875757B2 was substituted for 0.5 mL PureLink™ Plant RNA Reagent, samples were vortexed until homogenized in buffer, all centrifugation was performed at 16,000 × *g* at 4 °C, 200 μL of NaCl was used, 600 μL of chloroform was used for the first organic extraction, then the chloroform extraction was repeated using an equal volume of chloroform to aqueous layer (typically 1 mL), 3 × 1 mL ethanol washes were performed to improve sample purity and nucleic acid pellets were allowed to dry for 10 min before resuspension in 20 μL VWR molecular grade water.

DNA contamination was removed from RNA using Thermo Fisher DNAse1 (RNAse-free, catalog #EN0521) and the manufacturer’s protocol (Publication No. MAN0012000). After DNAse treatment, we further purified the RNA using a Zymo RNA Clean and Concentrator kit (Catalog #R1013; Irvine, CA) following the recommended protocol in the manufacturer’s manual. RNA was eluted in 15 μL RNAse-free water. Prior to sequencing, we determined final RNA concentration and integrity using an Agilent 4200 Tapestation System. Samples with less than 44 ng/μL and/or a RIN less than 5.0 were re-extracted.

Transcriptome sequencing was performed by The Pennsylvania State University Huck Institutes of the Life Sciences Genomics Core Facility. Lexogen QuantSeq libraries were created using the manufacturer’s protocol. Samples were then run in 5 batches, 32 samples per batch, on an Illumina NextSeq 550 in High Output mode with 75 bp reads, producing approximately 8 million reads per library.

### Genome meta-assembly

DNA from early-stage E leaves was extracted and sequenced according to previously outlined methods [41]. The linked read data for the *T. cacao* genotype SCA 6, as well as four other genotype, were assembled with Supernove v2.1 [42] at five raw read coverage depths of approximately 56x, 62x, 68x, 75x, and 85x based on the estimated genome sizes (Additional file 1: Table S5). We translated the Supernova assembly graph to create two parallel pseudohaplotype FASTA representations of the genome (pseudohap2 style) and utilized one pseudohaplotype from each of the five assemblies for subsequent post-processing. Among these five pseudohaplotype assemblies, we designated one of them as the optimum primary Supernova assembly using a combination of assembly metrics: completeness of annotated conserved land plant (embryophyta) single-copy BUSCO genes (Simão et al., 2015, Waterhouse et al., 2018), contig and scaffold contiguity (L50), and an assembly size closer to the estimated haploid genome size (Additional file 1: Tables S3-S5). Quickmerge [43] was then used to incrementally improve the backbone assembly by bridging gaps and joining contigs using the remaining four primary pseudohaplotype assemblies in decreasing order of assembly quality. After each merging step, the resulting meta-assembly was assessed for contiguity, completeness, and assembly size, only being retained if all three displayed improvement. Assembly errors introduced during de novo assembly and merging were corrected using the Tigmint [44] and ARCS [45] algorithms. Tigmint aligns linked reads to an assembly to identify potential errors, then breaks assembled sequences at the boundaries of these errors. The assembly is then re-scaffolded into highly contiguous sequences with ARCS utilizing the long-distance information contained in the linked reads. Gapfiller v1.10 [46] was used to iteratively fill gaps between contigs using paired-end reads from both the short insert Illumina libraries and the 10 × Chromium libraries. Finally, those same reads were used by Pilon v1.23 to correct base errors and local mis-assemblies.

### Pseudochromosome construction

Chloroplast, mitochondrial, and contaminant sequences present in the meta-assembly were removed prior to construction of the nuclear pseudochromosomes. To identify these extraneous DNA sequences, the meta-assembly was searched against the NCBI nucleotide collection database (*nt*) using Megablast [47]. Meta-assembly sequences with hits in the *nt* database were then queried against the NCBI taxonomy database to determine their taxonomic attribution. Meta-assembly sequences with best hits to non-embryophytes (land plants) were considered contaminants and discarded. We performed a second iteration of Megablast searches of the remaining meta-assembly sequences (embryophyte-only) against the NCBI RefSeq plant organelles database to identify chloroplast and mitochondrial sequences. Meta-assembly sequences with high similarity (> 80% identity and > 50% coverage) to sequences in the plant organelles database were also discarded. Finally, the remaining nuclear contigs and scaffolds were ordered and oriented into pseudomolecules with RaGOO [48] using the *T. cacao* L. cultivar Matina 1–6 v1.1 [49] reference chromosomes.

### Assembly evaluation and validation

We assessed the SCA 6 meta-assembly for contiguity, completeness, and structural accuracy by comparing it to the two published *Theobroma cacao* chromosome level reference assemblies of Matina 1-6 v2.1 and Criollo B97-61/B2 v2.0 [49, 50]. Both the contig and scaffold assembly metrics were evaluated in addition to completeness of universally conserved single-copy genes using the BUSCO land plants (embryophyta) benchmark gene set (Additional file 1: Table S4).

### Repeat library construction

Prior to annotation, repetitive and TE-rich regions of the genome were masked using the MAKER-P repeat masking protocol [51]. MITE-Hunter [52] and LTRharvest/LTRdigest [53, 54] were used to collect consensus miniature inverted-repeat transposable elements (MITEs) and long terminal repeat retrotransposons (LTRs) from the meta-assembly, respectively. LTRs were first filtered to remove elements with nested insertions, then combined with the MITEs to mask the genomes. The unmasked regions of the genomes were then annotated for de novo repetitive sequences using RepeatModeler1 (http://www.repeatmasker.org/RepeatModeler). Finally, all collected repetitive sequences were compared to a BLAST database of plant proteins from SwissProt and RefSeq, where proteins from transposable elements are excluded. Sequences with significant hits to the protein database were excluded from the repeat masking library, since these hits could be from authentic genes.

### Generation of gene annotation evidence

In order to capture robust transcript data to support genome annotation, we sequenced pooled RNA from variety of cacao tissue samples available in the Guiltinan-Maximova lab. Additional file 1: Table S6 provides information on tissue samples and experimental conditions including genotype, tissue type, developmental stage, growth conditions, and stress treatments. All harvested tissues were flash frozen in liquid nitrogen immediately on collection, homogenized to fine powder, and stored in liquid nitrogen or at −80 °C for RNA extraction. Total RNA was isolated from cacao tissue samples using Purelink Plant RNA Reagent following the same protocol outlined above. Extracted samples were cleaned by ethanol precipitation [55] before sample pooling. RNA extracted from tissue following salicylic acid treatment were collected and processed as previously described [40]. Individual and pooled RNA integrity was assessed on an Agilent 2100 Bioanalyzer System. Illumina TruSeq libraries (150 nt) were prepared using RNA pools at The Pennsylvania State University Huck Institutes of the Life Sciences Genomics Core Facility. Libraries were then sequenced on an Illumina NextSeq 550 in high output mode at the same facility.

Raw RNA-Seq reads were trimmed to remove low-quality bases as well as embedded adaptor sequences and filtered to discard short-read fragments using Trimmomatic v0.33 [56]. We then used FastQC v0.10.1 (https://www.bioinformatics.babraham.ac.uk/projects/fastqc/) to assess the overall sequence quality before and after trimming. Cleaned reads from each tissue sample were de novo assembled using Trinity [57] with the default parameters. The resulting transcriptome assemblies were post-processed with the PlantTribes2 AssemblyPostProcessor (https://github.com/dePamphilis/PlantTribes) to select contigs with potential coding regions to use as evidence for gene annotation.

### Gene prediction and functional assignment

Protein-coding gene annotations from the reference *T. cacao* genomes of Matina 1-6 v2.1 and Criollo B97-61/B2 v2.0 were separately transferred to pseudomolecules of the SCA 6 meta-assembly using the FLO (https://github.com/wurmlab/flo) pipeline, which is based on the UCSC Genome Browser Kent-Toolkit [58]. We then utilized the MAKER annotation pipeline (release 3.01.02) [59] to update transferred annotations with evidence data and to predict gene models with ab initio gene finders. Repetitive and low complexity regions of the pseudomolecules were first masked with RepeatMasker in MAKER using the previously described cacao-specific repeat library. The annotation evidence provided to MAKER includes previously described tissue- and stress-specific transcriptome assemblies. Additionally, predicted protein sequence from nine representative Malvid genomes, including *Gossypium raimodii*, *Gossypium hirsutum*, *Arabidopsis thaliana*, *Carica papaya*, *Citrus sinensis*, *Citrus clementina*, *Eucalyptus grandis*, *Panica granatum*, and *Populus trichocarpa* were provided as cross-species homology evidence. In the initial run of MAKER, transferred annotations were updated with evidence data and additional annotations were predicted with Augustus using a cacao training set. A second iteration of MAKER was performed using both Augustus and SNAP ab initio gene finders to further improve the quality of gene models [60, 61]. We selected approximately 5000 high confidence gene models from the initial MAKER run to train SNAP Hidden Markov models used to predict gene structure. MAKER only replaced a previously predicted gene model if annotation evidence suggested that a model from the second run was better. Complete functional annotation of gene sets was performed using the Blast2GO [62] functional annotation module. The best functional descriptors for gene products were assigned following BLASTp searches against the UniProt/SwissProt databases. Additionally, gene models were assigned to KEGG (http://www.kegg.jp/) pathways and annotated with protein family domains as detected by InterProScan [63]. Identified domains were directly translated into gene ontology terms.

### Expression quantification, differential expression, and gene ontology enrichment

Illumina 75-bp reads were trimmed to remove adapters using trimmomatic [56]. Reads were aligned to the genome using STAR [64] and quantified using featureCounts [65]. Differential expression analysis was performed using DESeq2 [66]. Due to variation in temperature, humidity, and leaf developmental stage across the experiment, we included both tray and leaf developmental stage as covariates in the model (Additional file 2: Fig S3). Moreover, because the experiment was unbalanced, i.e., containing inconsistent sample sizes both within and between phenotype classes and populations, we provided custom contrast matrices to DESeq2 for the differential expression calculations (Additional file 2: Fig S1). The contrast matrices add weights that help mitigate the bias introduced by differences in sample size and were calculated as follows:

### Treatment contrast

**Fig. 2:**
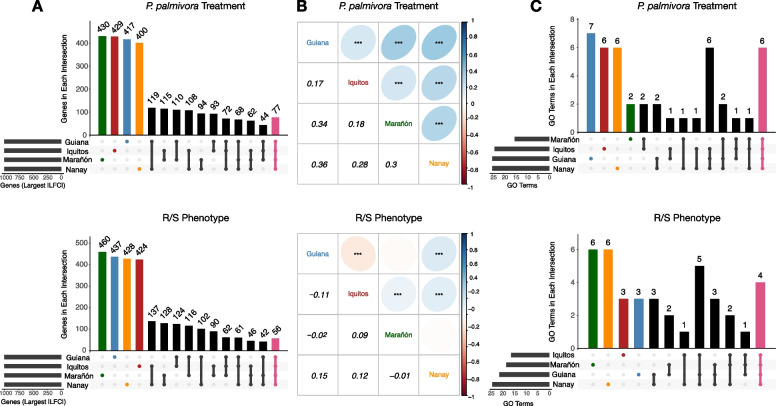
Different sets of genes are responsible for defense against *P. palmivora* across four cacao populations. **A** Overlap of differentially expressed genes for *P. palmivora* treatment versus control (top) and between resistant versus susceptible genotypes (bottom). The blue, red, green, and orange bars represent genes that are only differentially expressed in Guiana Iquitos, Marañón, or Nanay, respectively. The pink bar indicates genes that are differentially expressed across all four populations. Numbers above the bars indicate the number of differentially expressed genes in that specific intersection. **B** Pairwise Spearman correlations of log_2_ fold changes for all genes investigated in this study, for *P. palmivora* treatment versus control (top) and between resistant versus susceptible genotypes (bottom). The bottom off-diagonal is the Spearman correlation coefficient. The top off-diagonal is the correlation coefficient depicted as an ellipse, the shape of which depends on the size of the coefficient. Asterisks indicate statistical significance (*p* < 0.001), tested using Spearman’s rho. **C** Overlap of enriched GO terms (Fisher’s exact test: FDR-adjusted *p*-value < 0.05) for *P. palmivora* treatment versus control (top) and resistant versus susceptible genotypes (bottom). The blue, red, green, and orange bars represent GO terms that are only enriched in Guiana Iquitos, Marañón, or Nanay, respectively. The pink bar indicates GO terms that are significantly enriched across all four populations. Numbers above the bars indicate the number of enriched GO terms in that specific intersection

Treatment effects were calculated as the average log2 expression difference between treatment and control averaged over genotype. Phenotype effects were calculated as the average log2 expression difference between resistant and susceptible genotypes averaged over exposure. Interaction effects are the difference in treatment effects between resistant and susceptible, or equivalently, the difference in phenotype effects between treated and control. All effects were weighted by sample sizes within genotype to adjust for the imbalance in the design. Very few interaction effects were observed in our study, so we chose to omit them. After differential expression analysis, we chose the top 1000 genes ranked by absolute log2 fold change (LFC) to run gene ontology enrichment. We chose an arbitrary LFC cutoff, rather than one based on *p*-values after multiple test correction, because limitations in sample size and inter-genotype variation resulted in a loss of statistical power at the group level. To verify that our LFC cutoff did not cause spurious results (Fig. 2A), we performed the same analysis on two subsets of our data. First, we analyzed only those genes that were significantly differentially expressed (FDR-adjusted *p*-value < 0.05). Second, to verify that the large proportion of genes private to each population was not due to random chance, we compared the overlap of two types of subsamples. In the first type of subsample, we ranked the genes in each population by LFC before taking samples of size *N*, where *N* = 200–2000 genes. This protocol we used to choose the top 1000 differentially expressed genes. In the second type of subsample, gene sets were randomly sampled at size *N*, where *N* = 200–2000 genes. For both types of subsamples, we calculated the proportion of unique genes in each population, for each sized sample. We calculated whether differences in subsamples (LFC-ranked versus random subset) were significant using a one-way ANOVA followed by Tukey’s honest significant different (Tukey HSD).

Lastly, we verified that the genes unique to each population did not display significantly lower expression than the genes shared between populations (Additional file 2: Fig. S4). For both the treatment and phenotype main effects, the genes unique to specific populations were not systematically biased towards lower expression. In fact, for treatment effect, the genes unique to Guiana and Marañón had significantly higher expression than the genes shared among populations (one-way ANOVA, *p*-value < 2e − 16; Tukey’s HSD, FDR-adjusted *p*-value < 0.001). And for phenotype, the genes unique to Guiana, Marañón, and Nanay had significantly higher expression (one-way ANOVA, *p*-value < 2e − 16; Tukey’s HSD, FDR-adjusted *p*-value < 0.01).

We used the top 1000 genes, ranked by |LFC|, from each population for further analysis. We performed gene ontology (GO) enrichment analysis using topGO v2.38.1 (algorithm = “classic”, statistic = “fisher”), which produced a large list of enriched GO terms (FDR-adjusted *p*-value < 0.05). Because gene ontologies are organized as directed acyclic graphs (DAGs), leading to parent-child relationships between specific terms, GO enrichment methods often produce large, unwieldy lists that contain redundant information that complicates further analysis. Therefore, we exploited the structure of the DAGs to prioritize GO terms that lie close to the terminal leaves of the graphs using GOxploreR [67]. In this way, terms providing the most specific information were carried forward for further analysis. We then grouped similar GO terms using Lin’s measure of semantic similarity as implemented in REVIGO [68].

In order to determine whether each population was using different, yet evolutionarily related, genes to defend themselves against *P. palmivora*, we classified all predicted proteins in the SCA 6 genome into orthologous gene families. This was done using PlantTribes2 [69, 70], which employs a combination of BLAST [71] and hidden Markov models [72] to infer groups of genes that share a single common ancestor among a diverse set of 37 high-quality plant genomes (https://github.com/dePamphilis/PlantTribes).

### *TcCSE* cloning and overexpression in *Nicotiana benthamiana*

Cacao cDNA was prepared with DNaseI-treated RNA from stage A/B leaf tissue (cacao genotype SCA 6) using M-MuLV Reverse Transcriptase (NEB M0253S; New England Biolabs, Ipswich, MA, USA). *TcCSE* was cloned from cDNA using Phusion DNA Polymerase (NEB 0530S) and the primers TcCSE_for and TcCSE_rev (Additional file 1: Table S7). The primers introduced BsaI sites with overhangs 1 and 4 on the 5′ and 3′ end of the amplicon, respectively, for later subcloning into pGK19.0923 by Golden Gate assembly (see below). The amplicon was cloned into pMiniT 2.0 using the NEB PCR Cloning Kit (NEB E1202S) and verified by Sanger sequencing.

To facilitate rapid subcloning of *TcCSE* and other coding sequences into an overexpression vector, the binary vector pGZ12.0501 (GenBank KF871320.1) was converted into a GoldenGate assembly compatible vector [73, 74]. To achieve this, the PDK intron from pHANNIBAL (GenBank: AJ311872.1) was amplified by PCR (Phusion polymerase) with the primers PDK_BsaI_for and PDK_BsaI_rev. PDK_BsaI_for introduced one SpeI and two BsaI restriction sites on the 5′ end of the amplicon and PDK_BsaI_rev introduced two BsaI and one HpaI restriction sites on the 3′ end of the amplicon (Additional file 1: Table S7), resulting in the following amplicon with BsaI restriction sites with unique overhangs (in parentheses): (TGCC)/BsaI recognition site 1 (reversed) – BsaI recognition site 2/(GCAA) – PDK intron – (ACTA)/BsaI recognition site 3 (reversed) – BsaI recognition site 4/(TTAC). The amplicon was digested with SpeI and HpaI restriction enzymes and ligated into pGZ12.0501 between SpeI and HpaI sites using T4 DNA Ligase 4 (NEB M0202S). This resulting vector is referred to as pGK19.0923 was fully sequenced, annotated, and deposited in NIH Genbank (accession number OQ732918).

For Golden Gate assembly, pMiniT 2.0 plasmid harboring the *TcCSE* candidate coding sequences with BsaI adapters (sites 1 and 4) (~150 ng) was mixed with pGK19.0923 plasmid (~ 50 ng) in 1 × T4 DNA Ligase buffer (NEB B0202S), with T4 DNA Ligase (NEB M0202S, 200 U) and BsaI-HF-v2 (NEB R3733S, 10U) in a total reaction volume of 10 μl. The reaction mixture was incubated at 37 °C for 30 min, followed by 30 cycles of 37 °C (5 min)/16 °C (5 min), and a final heat denaturation at 60 °C (5 min). The product was transformed into *E. coli* (10-beta) for selection on LB-kanamycin plates. The resulting vector will be referred to as p35S:TcCSE and places the *TcCSE* coding sequence after the E12-Ω CaMV-35S constitutive promoter [75]. This vector is formally designated as pGK21.0402 was fully sequenced, annotated, and deposited in NIH Genbank (accession number OQ732917).

p35S:TcCSE and the backbone vector control pGH00.0126 (GenBank KF018690.1) [76] were transformed into *Agrobacterium tumefaciens* strain AGL1 [77] by electroporation. The *A. tumefaciens* cultures were grown overnight in liquid 523 media to an optical density (OD_600nm_) of ~1 as previously described [39]. Cells were pelleted by centrifugation (15 min at 5000 × *g*) and the cell pellet was resuspended in sterile water to an optical density (OD_600 nm_) of 0.4 ± 0.02 for *Nicotiana benthamiana* infiltration and transient expression.

Four volumes of *A. tumefaciens* culture harboring either the backbone vector or 35S:TcCSE constructs were mixed with one volume of p19 culture (*A. tumefaciens* with pDGB3alpha2_35S:P19:Tnos, Addgene #GB1203; Addgene, Watertown, MA, USA) [78] for co-infiltration.

*N. benthamiana* plants were grown to 4–5 weeks from seed. Stage 2 and 3 leaves, according to Ma et al. [79], were infiltrated with *A. tumefaciens* cultures on the abaxial side using a needle-less syringe as previously described [80].

At 48 and 96 h after infiltration, 1.5-cm (I.D.) holes were punched out using a cork borer from *N. benthamiana* leaf tissue expressing the GFP marker gene included in both pGH00.0126 and pGK19.0923 backbones. Two leaf discs from the same plant were placed in a 2-ml screw cap tube containing 1 ml of 80/20/0.1 methanol/water/formic acid (v/v/v) and constituted one sample. Samples were heated at 80 °C for 30 min. The supernatant was dried in a SpeedVac and the resulting pellet was dissolved in an equal volume of 90/10/0.1 water/methanol/formic acid (v/v/v), filtered (0.2 µm, nylon), and loaded into HPLC vials for LC-MS/MS analysis.

Samples were run in negative mode on an AB SCIEX 5600 Triple TOF with a Shimadzu Prominence UPLC at The Pennsylvania State University’s Metabolomics Core Facility at the Huck Institutes of the Life Sciences. We followed the instrument specifications previously outlined in Knollenberg et al. [55].

We analyzed spectral and separation data coming from the LC-MS/MS instrument using the XCMS v3.8.2 package in R v3.6.3. Feature detection was performed using the following parameters: ppm = 15, minimum peak width = 5, maximum peak width = 20, signal/noise threshold = 6, m/z diff = 0.01, integration method = 1, prefilter peaks = 3, prefilter intensity = 100, noise filter = 0. Peaks were then grouped according to the following parameters: bw = 5, minimum fraction = 0.4, m/z width = 0.015, minimum number samples = 1, maximum features = 100. An authentic standard of caffeic acid (Cayman Chemical) was used to identify the compound in cacao and *N. benthamiana* leaf extracts. For the untargeted LC-MS/MS analysis of the zoospore droplet assay, MS-DIAL v4.0 [81] was used to extract MS/MS spectra for the putative theobromine peak, which was compared to spectra obtained from MassBank Europe (https://massbank.eu/MassBank/) and MassBank of North America (https://mona.fiehnlab.ucdavis.edu/). See Additional file 1: Table S8.

### Plant metabolite extraction from selected transcriptome tissue samples

We extracted metabolites from leaf discs collected during the RNA-seq experiment (*Transcriptome experimental design and treatment*) as previously described [82, 83]. We flash froze leaf discs in liquid nitrogen and ground them in a mortar and pestle. Special care was taken to prevent the tissue from thawing. A 3:1 solvent to tissue ratio (µl:mg) was used to extract the metabolites, where the solvent was a solution of LC-MS/MS grade 80% methanol and 0.1% formic acid in water (v/v). Genistein was spiked into each sample to serve as an internal control [84]. Finally, we filtered residual particulates from the extract using spin columns (0.2 µm; Norgen Biotek Corp. Cat. #40000) before quantifying metabolites via LC-MS/MS. LC-MS/MS samples were again run using the specifications outlined in the previous section (“TcCSE cloning and overexpression in Nicotiana benthamiana”).

### *Phytophthora palmivora* growth inhibition and zoospore preparation

We performed growth inhibition assays to assess whether caffeic acid was capable of directly inhibiting *P. palmivora* strain Gh-ER1349 mycelial growth. First, pathogen cultures were taken out of storage in liquid nitrogen and grown on 20% V8 media [39] for 2 days. After 2 days, we sub-cultured the leading edge of the culture onto new plates with or without 2 mM caffeic acid. Plates were stored upside-down in the dark at 27 °C for 2 days, after which we determined mycelial growth inhibition using ImageJ [85]. We amended the plates with 2 mM caffeic acid because this concentration is on the low end of what has previously been considered physiologically relevant [86]. We prepared *P. palmivora* zoospores for the metabolite mobilization assay according to the following protocol. We prepared 125-mL Erlenmeyer flasks containing 25 mL V8 media. We placed two mycelial plugs in each flask and sealed them with foil and parafilm. In order to make sure pathogen cultures were kept in darkness, flasks were placed in a cardboard box in the incubator (27 °C) for 7 days. After 7 days, flasks were placed in 24-h light for 4 days, again at 27 °C. After this 11-day period, we induced zoospores by first flooding each flask with 25-mL sterile, ice-cold water. Flooded flasks were then placed in the refrigerator (4 °C) for 45 min before placing them back in the incubator (27 °C) for 30 min. We calculated the concentration of newly created zoospores using a hemocytometer. We resuspended zoospores in 50 mL Falcon tubes and immediately used them for experimentation. Zoospore suspension (25 µl; 50,000 spores/ml) was placed on the abaxial side of a SCA 6 stage C leaf and maintained at 25 °C and high humidity for 24 h. 15 µl from each drop (3 per leaf) were collected and pooled. Droplets from two leaves were collected this way and pooled to constitute one replicate. The solution was mixed 1:1 with methanol, filtered, and analyzed LC-MS/MS as above. A water “mock” inoculation was done in parallel, as well as a “zoospore only” control, which consisted of zoospore suspension on a sterile petri dish.

### Genome scan for selection

We searched for signals of selection at the genome level by using previously published short-read sequence data from the 31 genotypes [9]. After removing low-quality reads and sequencing adapters with Trimmomatic [56], we aligned the reads to the SCA 6 meta-assembly using BWA-MEM [87]. We removed duplicated reads with SAMtools [88] and called variable sites using BCFtools [89]. We only used reads with mapping- and base-quality ≥ 20 in the variant calling. The variant calls were then filtered to only keep biallelic SNPs with the following requirements: site- and genotype-quality ≥ 20, read coverage ≥ 6, < 20% missing data, and minor allele frequency > 0.05.

We used population branch statistics (PBS) [90] to estimate the genetic differentiation of lineages leading into the resistant genotypes of each population. Standard differentiation measures, such as F_ST_ or d_XY_, can detect signals of differential selection, but they generally cannot distinguish which of the populations has been the target of selection. To detect lineage-specific selection, PBS uses an outgroup to polarize differentiation measures between two closely related populations. Assuming a closely related population pair 1 and 2, and an outgroup 3, PBS for population 1 is estimated as:

$${\text{PBS}}_{1}=\frac{{{\text{T}}}_{12}+{{\text{T}}}_{13}-{{\text{T}}}_{23}}{2},$$

where *T* is the relative divergence time, *T* = − ln(1 − Fst), estimated for each pair of populations. Using the F_ST_ estimator by Hudson 1992 [91], we first quantified differentiation between the resistant and susceptible genotypes of each population. Then, to find selection acting on the resistant class, we combined the susceptible genotypes from the three remaining populations to act as an outgroup. The reasoning behind this approach is that alleles responding to pathogen-mediated selection in the resistant genotypes should be either neutral or deleterious in the susceptible genotypes, revealing longer-than-expected branch lengths leading into the resistant lineages. To better associate the selection signals with results from the transcriptome experiment, we estimated PBS specifically for each gene, including the surrounding regulatory regions. Consistent with previously published methods [92–94], we categorized the top 1% of PBS scores as selection outliers, highlighting those that were also differentially expressed (Additional file 1: Tables S9-S10).

### Transcriptome experiment for non-cacao *Theobroma* species

The transcriptome experiment followed a split-plot design, where tree was treated as the blocking factor. Over three consecutive days, we sampled leaves from a single tree for each species (*N* = 3 trees per species). From each tree, we took four leaves, two for *P. palmivora* treatment and two for controls. These leaves were pooled to create a single biological replicate (*N* = 3 treatment samples, *N* = 3 control samples per species). Care was taken to select leaves that did not display any visible signs of damage or pathogen infection. Because leaf developmental timeline is less well-characterized for non-cacao *Theobroma spp.* than it is for *T. cacao*, we used fully mature leaves. Leaves were treated with either *P. palmivora* plugs or control plugs as previously described [36]. Within each tree, we randomized the order in which we processed each species. A cork borer was used to punch out leaf discs surrounding the necrotic lesion area 48 h post inoculation, or, for the controls, leaf discs surrounding the agar plug. Leaf discs were then put into 2-mL cryovial tubes and flash frozen using liquid nitrogen.

RNA was extracted according to the protocol outlined in the previous section. Library construction and sequencing were done at The Pennsylvania State University Huck Institutes of the Life Sciences Genomics Core Facility. Stranded, single end, 150 nt libraries were sequenced on two high output runs of an Illumina NextSeq 550. This generated approximately 30 million reads per sample and approximately 200 million reads per species.

### Transcriptome analysis for non-cacao *Theobroma* species

Adapters and low-quality bases were trimmed from the reads using Trimmomatic v0.38 (SE -phred33 ILLUMINACLIP:TruSeq3-SE:2:30:10 LEADING:3 SLIDINGWINDOW:4:15 MINLEN:50) [56]. Reads were then assembled into transcripts using Trinity v2.11.0 (--seqtype fq --single --SS_lib_type R --no_normalize_reads --no_cleanup --bflyHeapSpaceMax 20) [57]. Transcripts were post-processed into putative coding sequences and their corresponding amino acids using TransDecoder (https://github.com/TransDecoder/TransDecoder) as implemented in the PlantTribes2 AssemblyPostProcessor pipeline [69, 70]. Non-embyrophyte contaminants were then cleaned from predicted coding sequences using a BLAST-based procedure. First, predicted coding sequences were searched against the NCBI nonredundant (*nr*) database. The BLAST hits were then queried against NCBI’s taxonomy database to assign taxonomic class. Finally, assembled sequences with top hits outside embryophyta (land plants) were discarded using a custom set of Bash and Perl scripts.

For a variety of reasons, including RNA degradation, genome heterozygosity, alternative splicing, etc., transcriptome assemblies are often highly fragmented [95]. This can lead to multiple assembled transcripts originating from the same gene, which can cause redundant read mapping and inappropriate expression quantification. To address this problem, we created “supertranscripts” by generating consensus sequences from transcripts belonging to the same Trinity cluster [95]. We first separated predicted coding sequences and amino acids by clusters, i.e., transcripts possessing identical IDs other than the isoform suffix. We then aligned each cluster of amino acid sequences with MAFFT v7.20 (L-INS-i) [96]. The coding sequences were then forced onto these amino acid alignments to create codon alignments. From each cluster, a set of hidden Markov models (HMM) were created from the amino acid and coding sequence alignments using HMMER v3.1b1 [72]. The majority-rule consensus (> 50%) sequence was then called from each HMM using hmmemit (-c -o). This consensus sequence represents a cluster’s putative supertranscript. Finally, to remove premature stop codons and other potential artifacts that may have been introduced during supertranscript construction, putative supertranscripts were cleaned using the PlantTribes2 PostAssemblyProcessor [70].

Supertranscript abundance was quantified using Kallisto (-i -o -b 100 –single -l 200 -s 20 -t 5) [97] and plugged directly into limma voom [98] for differential expression analysis. Our experiment was implemented as a split-plot design with tree as a blocking factor. An unadjusted *p*-value cutoff of 0.05 was used to define supertranscripts as differentially expressed. We used unadjusted *p*-values rather than *p*-values corrected for multiple testing for two reasons. First, we were primarily interested in using the differential expression results to identify groups of orthologous genes that were responding consistently across species, rather than identify specific genes that may be important for disease resistance. When looking at sets of aggregated genes, we are less worried about multiple test correction, since it is unlikely that we would observe an orthogroup that is differentially expressed across multiple species due to false positives alone. Second, our small sample size and large standard error made FDR-adjusted *p*-values > 0.05 for most supertranscripts [99].

### Analysis of log_2_ fold change across species

To gain a better understanding of how defense response evolved in *Theobroma*, and to better predict groups of genes that may be important for resistance specifically in *T. cacao*, we compared orthogroup expression from the *T. cacao* transcriptome results to our non-cacao *Theobroma spp*. Mean orthogroup log_2_ fold change (LFC) for each *Theobroma spp.* was compared to mean LFC across all populations of *T. cacao*. Differentially expressed orthogroups that were strongly responsive (|LFC|> 1), shared across all four non-cacao *Theobroma spp.*, i.e., core, *and* also differentially expressed in at least one *T. cacao* population, were labeled as “core & |LFC|> 1” and carried forward for further analysis (Additional file 1: Table S11).

### Branch-site tests of positive selection

We tested whether core orthogroups with mean |LFC|> 1 (*N* = 48) were evolving under diversifying selection using HyPhy’s branch-site unrestricted statistical test for episodic diversification (BUSTED) (--alignment --tree --branches --output). BUSTED is a branch-site method that, given a set of foreground and background branches, tests whether a subset of codons in a gene have undergone positive selection [100]. We began by classifying all supertranscripts predicted during transcriptome assembly into orthogroups, as described above. From each orthogroup, we extracted sequences for all *Theobroma spp.*, as well as a subset of the species used for classification: *Elaeis guineensis* (Arecaceae), *Oryza sativa* (Poaceae), *Lactuca sativa* (Asteraceae), *Solanum lycopersicum* (Solanaceae), *Arabidopsis thaliana* (Brassicaceae), *Theobroma cacao* (Malvaceae), *Medicago truncatula* (Fabaceae), *Vitis vinifera* (Vitaceae), *Aquilegia coerulea* (Ranunculaceae), *Amborella trichopoda* (Amborellaceae). We then aligned each orthogroup at the amino acid level using the MAFFT v7.205 L-INS-I algorithm, unless a gene family was > 1000 sequences, in which case --auto was used [96]. The coding sequences were then forced onto the amino acids to create a codon alignment using a custom Perl script. To improve codon alignments, we trimmed columns that were primarily composed of gaps using TrimAl (-gappyout) [101], and completely removed sequences that were composed of > 70% gaps. Trees were built from each orthogroup alignment using FastTree v2.1.10 (-nt -gtr) [102]. Finally, BUSTED models were implemented using HyPhy [103]. All *Theobroma spp.*, including *T. cacao*, were used as the foreground while all other species were used as background.

## Results

### Meta-assembly of the cacao SCA 6 genome

There are currently two reference genomes for cacao: the rare, fine-flavor cacao genotype Criollo B97–61/*B2* [50, 104], and one of the most widely cultivated cacao genotypes, Matina 1-6 [49]. Neither genotype is highly resistant to *Phytophthora spp*. Therefore, the extent to which Criollo and Matina can be used to identify candidate resistance genes in a diverse set of cacao genotypes is unclear. To facilitate identification of novel resistance genes, we de novo assembled and annotated the *Phytophthora-*resistant genotype Scavina 6 (SCA 6). SCA 6 does not belong to any of the four populations sampled for this study and is thus unlikely to bias mapping rates due to relatedness. We assembled SCA 6 from 10X Genomics linked read technology [105] using a novel meta-assembly approach (“Methods”) that created separate assemblies at multiple read depths, followed by iterative bridging between assemblies. This resulted in a highly contiguous, near-reference level genome (Scaffold N50 (Mb): 2.344; Contig N50 (Kb) 245.957), with BUSCO genome assembly completeness scores (97.2%) that indicated that most of the gene space was captured (Additional file 1: Table S4).

### Cacao genotypes and populations sampled for this study

We selected 31 cacao genotypes based on previously described levels of resistance to the black pod rot pathogen *Phytophthora palmivora* [36]. Each genotype belongs to one of four populations named for their original geographic location [14] in the Amazon basin: Guiana, Iquitos, Marañón, or Nanay (Fig. 1A, B). Based on whole-genome sequence data, these four populations are genetically distinct and bear some evidence of local adaptation [9, 41]. From a subset of each population that was previously phenotyped for resistance [36], we chose the four most resistant and four most susceptible individuals for further experimentation, with the exception of Nanay from which we had access to four susceptible but only three resistant genotypes. The resistant genotypes were as follows: (Guiana) Ker 1L, GU 257E, Pina, and OYA 2B; (Iquitos) IMC 60, COCA 3370/5, SPEC 54/1, and Amaz 15/15; (Marañón) NA 246, PA 13, PA 16, and PA 279; (Nanay) NA 7/10, Pound 7, and NA 916. The susceptible genotypes were as follows: (Guiana) ELP 37A, GU 123 V, GU 195 V, and Ker 6; (Iquitos) Amaz 12, IMC 105, IMC 31, IMC 57; (Marañón) PA 107, PA 299, PA 81, and PA 71; (Nanay) NA 70, NA 807, NA 33, and NA 34.

To investigate how divergence among populations affects the evolution of cacao’s defense response, and to discover potentially novel mechanisms underlying defense to *P. palmivora*, we performed an RNA-seq experiment. We began by importing 31 genotypes as grafted plants from the ex situ International Cocoa Collection (IC3) at the Tropical Agricultural Research and Higher Education Center (CATIE), Costa Rica. Approximately 300 cuttings were taken from these grafted plants, of which 141 rooted plants survived, representing 27 genotypes. The healthy plants were established in the greenhouse at Penn State University (Additional file 1: Table S12). To minimize the effects of greenhouse gradients in temperature, humidity, and other abiotic factors, 6-week-old plants were distributed across the greenhouse in a pseudo-randomized block design (Additional file 2: Fig. S2). Individual leaves on each plant were challenged with multiple agar plugs of *P. palmivora* or mock inoculant and samples were collected at 8 h post inoculation. This 8-h period was chosen based on preliminary experiments (unpublished data) to detect early defense regulation and transcriptional changes in specific defense-associated genes prior to extensive necrosis.

### Different sets of genes are responsible for defense against *P. palmivora* across the four populations

RNA from 141 samples was sequenced (Additional file 1: Table S12), producing an average of 8 million QuantSeq (see “Methods”) reads per library, of which approximately 80% mapped to SCA 6. Because 3′ tagging methods like QuantSeq produce a single read per transcript, even low coverage QuantSeq libraries can capture moderately expressed genes [106]. After testing for differential expression using DESeq2, the top 1000 genes ranked by absolute log_2_ fold change (LFC, hereafter referred to as differentially expressed genes—DEGs) were analyzed further. We examined two types of transcriptional response, hereafter referred to as main effects: response to pathogen treatment (hereafter treatment) and differences between resistant/susceptible (R/S) phenotypes (hereafter R/S phenotype). Treatment X phenotype interaction effects were weak and rare across all populations (total *N* = 37) and were therefore omitted from subsequent analysis. For each of our main effects, we started by examining the proportion of DEGs that were shared across populations. Of the 1000 DEGs chosen from each population (Additional file 1: Tables S13-S14), over 40% were in only one of the four populations (Treatment: Mean_% unique_ = 41.9, SEM_% unique_ = 0.7; Phenotype: Mean_% unique_ = 43.7, SEM_% unique_ = 0.8; Fig. 2A). Moreover, not only were many of the DEGs from each population unique, LFC correlations among all expressed genes (approximately 17 k) revealed inconsistent responses (Fig. 2B). This reveals that genes across all four populations responded differently to both pathogen challenge and R/S phenotype.

To verify that our LFC cutoff did not bias interpretation of the results, we performed the same analysis on two subsets of our data. First, we examined the effect of using a traditional, adjusted *p*-value cutoff [99]. We observed a larger proportion of DEGs that were unique to each population, for both pathogen treatment (Mean_% unique_ = 55.3, SEM_% unique_ = 14.9) and R/S phenotype (Mean_% unique_ = 68.6, SEM_% unique_ = 7.3). Second, we examined the effect of using different sized gene set cutoffs, ranging from 200 to 2000 genes. For each sample size, the proportion of DEGs that were uniquely expressed in each population ranged from 30 to 40%. While this proportion is higher than we see among other closely related individuals [107], it was still significantly lower than if the genes were selected at random (Additional file 2: Fig. S5; *p* < 0.001). Hence, the high degree of population uniqueness was not due to size of the subset or random chance.

Recent gene duplications can result in highly similar copies of the same gene. If populations are expressing different, yet closely related, copies (paralogs) of the same genes in response to *P. palmivora*, our observation that population responses were largely non-overlapping may be inflated. To test whether closely related genes were behaving similarly across populations, we clustered paralogs using a 95% identity cutoff. We then calculated the proportion of paralogous clusters that were unique to a given population or shared across populations. For both the pathogen treatment and R/S phenotype main effects, this resulted in patterns very similar to those in Fig. 1 (Treatment: Mean_% unique_ = 40.1, SEM_% unique_ = 0.6; Phenotype: Mean_% unique_ = 41.9, SEM_% unique_ = 1.0; Additional file 2: Fig. S6). Therefore, differences in DEGs among populations did not seem to be inflated by the differential expression of closely related paralogs.

To investigate the potential for functional redundancy in less closely related paralogs, we classified genes into orthogroups, i.e., narrowly defined protein families inferred to have a single ancestral gene among the species being compared [69, 70, 108]. We then calculated the proportion of differentially expressed orthogroups that were unique to each population (Additional file 2: Fig. S7). Orthogroups containing one or more DEGs were considered differentially expressed orthogroups. We found a smaller proportion of differentially expressed orthogroups unique to each population than we found when examining individual genes, for both pathogen treatment (Mean_% unique_ = 28.9, SEM_% unique_ = 1.2; *t*-test, *p*-value < 0.001) and R/S phenotype (Mean_% unique_ = 32.3, SEM_% unique_ = 2.6; *t*-test, *p*-value < 0.05). Average LFC among orthogroups, however, was again only weakly correlated across populations (Additional file 2: Fig. S8). Thus, each population used different, but often evolutionarily related genes to respond to *P. palmivora*.

Few studies have examined defense response across many genotypes from multiple populations. However, our results contrast at least one recent study in *Arabidopsis*, wherein the evolution of immunity-related gene expression was tested by treating *A. thaliana* and its close relatives with the microbial elicitor flg22. Of the genes differentially expressed in response to flg22, the proportion of 1:1 orthologs unique to each species was approximately 20–31% [107]. When their focus was limited to solely *A. thaliana* genotypes, the proportion of genes private to each genotype decreased even further, falling to approximately 3.5–12.5%. Moreover, average LFC correlations between differentially expressed 1:1 orthologs, both between and within species, were considerably higher than we observed among cacao populations (between *A. thaliana* and other Brassica, Mean_cor. coef._ = 0.73 SEM_cor. coef._ = 0.004; within *A. thaliana*, Mean_cor. coef._ = 0.88, SEM_cor. coef._ = 0.009; Additional file 2: Fig. S9). These results suggest potentially strong differences in defense response among populations and underline the need for further comparative work to evaluate variation both within and among taxa. Lastly, there were nearly 350 genes differentially expressed in our study that did not possess 1:1 orthologs in the Criollo or Matina genomes. Many of these genes had annotations associated with defense response, including WRKY transcription factors and pattern recognition receptors. Thus, it appears assembling, annotating, and using the SCA-6 genome for this manuscript helped capture genes that would have otherwise been impossible to identify.

### Common functional groups are shared by different sets of pathogen-responsive genes

**Fig. 3:**
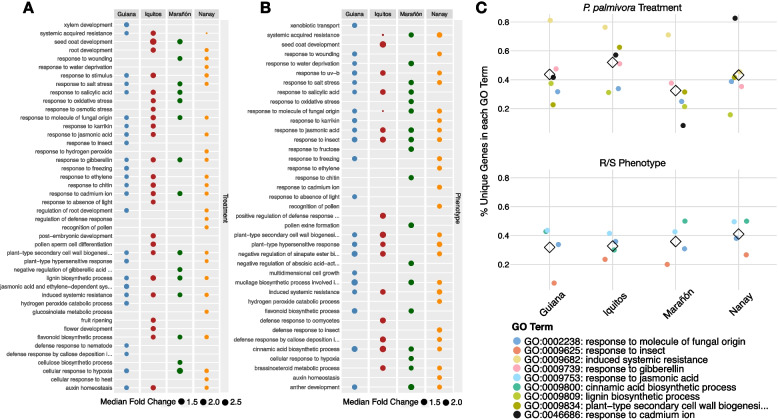
Common functional groups underlie different sets of pathogen-responsive genes. **A** Enriched GO terms (Fisher’s exact test: FDR-adjusted *p*-value < 0.05) and their median fold change for *P. palmivora* treatment versus control. Colored points indicate population membership: Guiana (blue), Iquitos (red), Marañón (green), or Nanay (orange). Point size is scaled to median fold change for the differentially expressed genes belonging to that term. **B** Enriched GO terms (Fisher’s exact test: FDR-adjusted *p*-value < 0.05) and their median fold change for resistant versus susceptible genotypes. Colored points indicate population membership: Guiana (blue), Iquitos (red), Marañón (green), or Nanay (orange). Point size is scaled to median fold change for the differentially expressed genes belonging to that term. **C** The percentage of genes from each population that are unique, calculated for each GO term that is enriched in all four populations. Terms that are significantly enriched in *P. palmivora* treatment versus control are on top, and terms that are significantly enriched in resistant versus susceptible genotypes are on bottom. Each point represents the proportion of differentially expressed genes belonging to a single GO term (indicated by color) that are unique to each population. For instance, Guiana has 22 differentially expressed genes in GO:0009834, 5 of which are not differentially expressed in any other population (5/22 = 22.7%). Means are shown as open diamonds

The large number of genes unique to each population does not preclude overlapping functional response. We compared functional similarity among our DEGs, either in response to pathogen challenge or between R/S phenotype, using gene ontology (GO) terms (Fig. 3A and B). There were more shared GO terms than individual genes (Treatment: Mean_% unique_ = 22.6, SEM_% unique_ = 3.2; Phenotype: Mean_% unique_ = 22.8, SEM_% unique_ = 4.1; Fig. 2C), suggesting many of the defense-related genes in each population belong to shared functional responses. Even the GO terms that were unique to each population often shared similarity, e.g., “response to auxin” and “auxin homeostasis”. While we tried to reduce redundant GO terms by exploiting the parent-child structure of GO directed acyclic graphs, some partially overlapping terms remained. Thus, the proportion of functional categories that were private to each population was likely lower than estimated above.

The list of GO terms significantly enriched across all populations contains some well-known defense-related processes. For the pathogen treatment main effect, these included “response to molecule of fungal origin” (GO:0002238), “induced systemic resistance” (GO:0009682), “response to gibberellin” (GO:0009739), “lignin biosynthetic process” (GO:0009809), “plant-type secondary cell wall biogenesis” (GO:0009834), and “response to cadmium ion” (GO:0046686). For the R/S phenotype main effect, we saw “response to molecule of fungal origin” (GO:0002238), “response to insect” (GO:0009625), “response to jasmonic acid” (GO0009753), and “cinnamic acid biosynthetic process” (GO:0009800). Even within this limited set of GO terms, however, 30–40% of the genes responding in each population were unique (Fig. 3C). This mirrors the pattern observed when examining all differentially expressed genes (Fig. 2A). Thus, even within this small, conserved subset of cacao’s defense response, many genes within each population responded uniquely.

The genes shared across populations included a cast of well-known defense mediators. Those responding to pathogen treatment across all four populations included multiple WRKY transcription factors [109, 110], as well as chitinase and endochitinase genes [111, 112]. Less well-known, but strongly upregulated, defense mediators included Gretchen Hagen3 (GH3) and multiple berberine bridge enzymes (BBE) [113–116]. Likewise, there were also several well-known defense regulators among the genes differing between R/S phenotypes across all four populations. These included a serine-threonine protein kinase (putative LRK10), a nucleotide-binding leucine-rich repeat protein (NLR), and several lipoxygenase enzymes, all of which represent protein families with well-known roles in pathogen detection, signal transduction, and subsequent defense [117–119]. Lastly, we also observed many genes involved in the formation of metabolites derived from the phenylpropanoid pathway, such as flavonoids, lignins, and hydroxycinnamic acids, all well-known components of plant defense responses. Among these were flavin-dependent mono-oxygenases, caffeic acid 3-O-methyltransferases, hydroxycinnamoyltransferses, and caffeoyl shikimate esterase (*TcCSE*) [120–123].

**Fig. 4:**
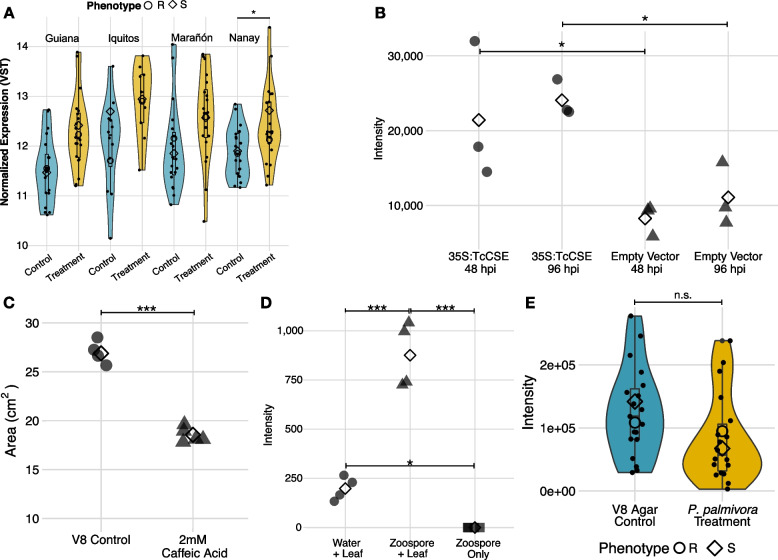
*TcCSE* is involved in resistance of *T. cacao* to *P. palmivora. ***A** Expression of *TcCSE* (SCA6_Chr6v1_17513) across each population for control (blue) and treatment (yellow). In each population, expression is consistently higher after treatment. However, the difference in gene expression between control samples and treatment samples was only significant in the Nanay population (FDR-adjusted *p*-value < 0.05). Open diamonds indicate mean expression for susceptible genotypes and open circles indicate mean expression for resistant genotypes. The top and bottom of the box and whisker plots represent the 75th and 25th percentiles, respectively. Whiskers represent 1.5 times the interquartile range. **B** Relative abundance (intensity) of caffeic acid in *N. benthamiana* plants transformed with p35s:TcCSE or a control backbone (“empty”) vector control, at both 48 and 96 h post transformation. Means are shown as open triangles. Over-expression of *TcCSE* results in significantly higher caffeic acid accumulation relative to controls (*t*-test 48 hpi: *p*-value = 0.0164; *t*-test 96 hpi: *p*-value = 0.0174). **C** Mycelial area of *P. palmivora* cultures grown on plates of V8 media versus plates of V8 media amended with 2 mM caffeic acid. Means are shown as open triangles. Plates amended with 2 mM caffeic acid significantly inhibited mycelial growth (*t*-test: *p*-value < 0.001). **D** Relative abundance of theobromine from water (“mock”) or *P. palmivora* zoospore droplets placed on cacao leaves, or zoospores only (not in contact with leaf). Means are shown as open diamonds. Cacao leaves challenged with zoospores accumulated significantly more theobromine than either mock inoculated or zoospore-only controls (*t*-tests: *p* < 0.001). Mock inoculated leaves had significantly more theobromine than zoospore-only controls (*t*-test: *p*-value = 0.022). **E** Relative abundance of caffeic acid in samples challenged with plugs of V8 media (blue) versus plugs of *P. palmivora* mycelia (yellow). There were no significant differences between treatment, phenotype, or the treatment:phenotype interaction (one-way ANOVA, Intensity ~ Treatment + Phenotype + Treatment:Phenotype: *p* > 0.05)

This set of differentially expressed metabolic genes suggests potential involvement of a diverse array of secondary metabolites, some of which are likely antimicrobial. *TcCSE* (SCA6_Chr6v1_17513), the 1:1 ortholog of *AtCSE* (AT1G52760.1), stood out as a particularly attractive experimental candidate for several reasons. First, *TcCSE* was consistently upregulated in response to pathogen challenge across all four populations (Fig. 4A). Second, *TcCSE* is a member of the phenylpropanoid pathway and, in *Arabidopsis*, is responsible for hydrolyzing caffeoyl shikimate into the hydroxycinnamic acid (HCA) caffeate (caffeic acid) [124]. HCAs and derivatives thereof are well-known antimicrobial secondary metabolites involved in various plant-pathogen interactions [83, 86, 125–127]. Together, these results indicate *TcCSE* could be a potentially important and as yet uncharacterized gene involved in cacao’s defense response. Accordingly, we performed a series of functional experiments involving *TcCSE*, both to verify our candidate gene identification approach and to evaluate this particular gene as a potential breeding target.

### Functional analysis of a candidate gene for caffeic acid synthesis

To begin characterizing *TcCSE*’s role in cacao’s defense response, we first verified its function through transient, heterologous overexpression in *N. benthamiana*. To accomplish this, we cloned *TcCSE* from the SCA 6 variety of cacao driven by a E12-Ω CaMV-35S constitutive promoter. Using Agrobacterium-mediated transformation, *N. benthamiana* plants were inoculated with *35S:TcCSE* vector or the corresponding backbone (“empty”) vector control. Consistent with its documented function in *Arabidopsis*, transient overexpression of *TcCSE* resulted in significantly higher caffeic acid accumulation relative to our backbone vector control (*t*-test 48hpi: *p*-value = 0.0164; *t*-test 96hpi: *p*-value = 0.0174; Fig. 4B).

While caffeic acid directly inhibits *P. palmivora* zoospore germination [86], its inhibitory effects towards mycelia growth have not been tested. Moreover, it remains unclear whether caffeic acid is directly inhibitory in planta. To address these points, we performed two experiments. First, we cultured *P. palmivora* on plates with or without 2 mM caffeic acid. As expected, including 2 mM caffeic acid in the media contributed to significant inhibition of mycelial growth (*t*-test: *p*-value < 0.001; Fig. 4C). Second, to determine whether caffeic acid or derivatives are mobilized to the site of infection, which is necessary for direct contact and subsequent inhibition, we placed water droplets containing *P. palmivora* zoospores on detached cacao leaves. After 24 h, the droplets were collected and analyzed using LC-MS/MS. In other work, a multidrug and toxin extrusion (MATE) transporter from *Arabidopsis thaliana* was shown to secrete antimicrobial hydroxycinnamic acid amides (HCAAs), including caffeic acid derivatives, into water droplet suspensions of *P. infestans* zoospores on the leaf surface, thereby preventing colonization [128]. Here, neither caffeic acid nor any of the caffeic acid-derived HCAAs reported in cacao leaf by Knollenberg et al. [55] were detected in the zoospore droplet, mock water control, or zoospore only (not on leaf) control. Unexpectedly, a signal consistent with theobromine (m/z [M-H]^−^ = 179.0574) was detected in the water mock inoculation and the zoospore droplet on the leaf surface. It was 4.4-fold higher in the zoospore droplet than the mock water control (*t*-tests: *p* < 0.001; Fig. 4D), and significantly higher in the mock water control than in the zoospore only control (*t*-test: *p*-value = 0.022). Comparing the compound’s mass spectrum to that of theobromine from a database (MassBank of North America LU094156) revealed mass congruence (< 0.0001 Da difference) of the parent ion and nine shared MS/MS fragments within 0.021 Da (Additional file 1: Table S8). Theobromine accumulation outside of the plant cell at the zoospore-leaf interface may play a yet unexplored role in defense, especially considering that theobromine and caffeine, the two most abundant methylxanthines in cacao, inhibit the in vitro growth of *Moniliophthora perniciosa*, another cacao pathogen [129]. Database spectra of two isomers of theobromine, paraxanthine, and theophylline, also shared several MS/MS fragments with the compound in question (Additional file 1: Table S8). These annotations cannot be ruled out without further characterization.

We next used LC-MS/MS to test the hypothesis that cacao plants with higher *TcCSE* expression had higher levels of caffeic acid 8 h after challenge with *P. palmivora* mycelia (Fig. 4E, Additional file 1: Table S15). There were no significant differences between treatment, phenotype, or the treatment X phenotype interaction (one-way ANOVA, Caffeic Acid Intensity ~ Treatment + Phenotype + Treatment X Phenotype: *p* > 0.05). This result did not support our initial hypothesis, but as we elaborate in the discussion, sampling one metabolite at one time point may not have been sufficient to characterize the relevant phenotype.

### Population branch statistics identify differentially expressed genes under selection

**Fig. 5:**
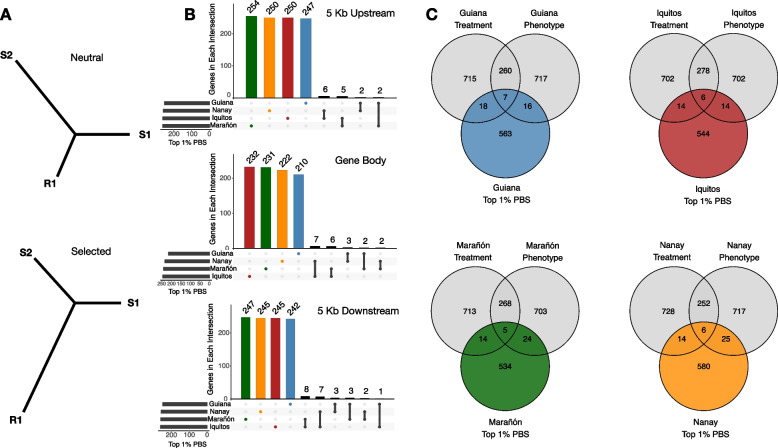
Population branch statistics identify differentially expressed genes under selection. **A** Population branch statistics can estimate lineage-specific selection leading to resistant genotypes. Branch lengths represent the magnitude of allele frequency change. For loci evolving neutrally in both resistant and susceptible genotypes, differences in allele frequency between resistant and susceptible individuals of the same population (S1, R1) will be *smaller* than allele frequency differences between susceptible individuals from two separate populations (S1, S2) (top). For loci under selection in resistant genotypes, differences in allele frequency between resistant and susceptible individuals of the same population (S1, R1) will be *greater* than allele frequency differences between susceptible individuals from two separate populations (S1, S2) (bottom). High PBS scores indicate genes that are under selection among resistant individuals from a given population. **B** Overlap of genic and non-genic regions designated as selection candidates (top 1% of their respective PBS distributions). PBS was estimated for 5 kb upstream of each gene (top), the gene body (middle), and 5 kb downstream of each gene (bottom). The blue, red, green, and orange bars represent genes that are only designated as selection outliers in Guiana, Iquitos, Marañón, or Nanay, respectively. Numbers above the bars indicate the number of selection outliers in that specific intersection. For all three regions, selection candidates are almost exclusively found in a single population. **C** Venn diagrams displaying the overlap between differentially expressed genes and genes under selection in resistant genotypes. Colors indicate population membership: blue (Guiana), red (Iquitos), green (Marañón), and orange (Nanay). Differentially expressed genes that are under selection in resistant individuals from a given population (intersection of the Venn diagrams) are high-quality candidates for further experimentation

Many of the DEGs detected in our transcriptome experiment, both in response to pathogen challenge and between R/S phenotypes, were unique to each population (Fig. 1A). This suggests that at least some aspect of each population’s defense response against *P. palmivora* is lineage-specific and that resistance versus susceptibility may be mediated by different genes depending on the population. This supports our original hypothesis that each wild population adapts to its environment, potentially generating a rich source of novel alleles. To determine the extent to which natural selection has shaped resistance and susceptibility in each population, we used population branch statistics (PBS) to estimate the lineage-specific genetic differentiation associated with resistant genotypes in each population (Fig. 5A) [90]. We estimated PBS for the coding region of each gene, as well as 5 kb on both the 5′ (hereafter upstream) and the 3′ ends (hereafter downstream). Thus, each gene has three PBS values. Genic and non-genic regions in the top 1% of their respective PBS distributions were considered selection candidates. Across the four populations, this candidate cutoff resulted in 1016 5 kb upstream candidates, 915 coding region candidates, and 1003 in the 5 kb downstream region (Fig. 5B).

The vast majority of PBS candidate genes are unique to each population. This pattern is similar to that observed among the differentially expressed genes, which again suggests defense responses are often population specific. Among these selection candidates, 163 were also differentially expressed in response to pathogen challenge, R/S phenotype, or both (Fig. 5C). Moreover, many of these genes can also be found within the limited set of GO terms shared across all populations, including the cinnamic acid biosynthetic process, induced systemic resistance, response to gibberellin, response to jasmonic acid, and response to molecule of fungal origin. Three of the genes defined as selection candidates are differentially expressed across all four populations: *TcWRKY29* (SCA6_Chr3v1_10161, pathogen treatment), *TcBBE8* (SCA6_Chr6v1_16921, pathogen treatment), and *TcFMO1* (SCA6_Chr9v1_23321, pathogen treatment and R/S phenotype) (Additional file 1: Tables S13-S14). The fact that these three genes are differentially expressed, present in the small number of GO terms enriched across all four populations, and show signatures of divergence among resistant genotypes makes them highly attractive candidates for future experimentation.

### Transcriptome responses in non-cacao *Theobroma* species reveal orthologous defenses

While the differential expression results suggest that each population employs a distinct set of genes in response to *P. palmivora* challenge, certain aspects of their defenses are consistent. This indicates that some portion of cacao’s defense against *P. palmivora* is mediated by orthologous genes, i.e., genes that arose prior to the separation of these four populations, and potentially even predate cacao speciation.

**Fig. 6:**
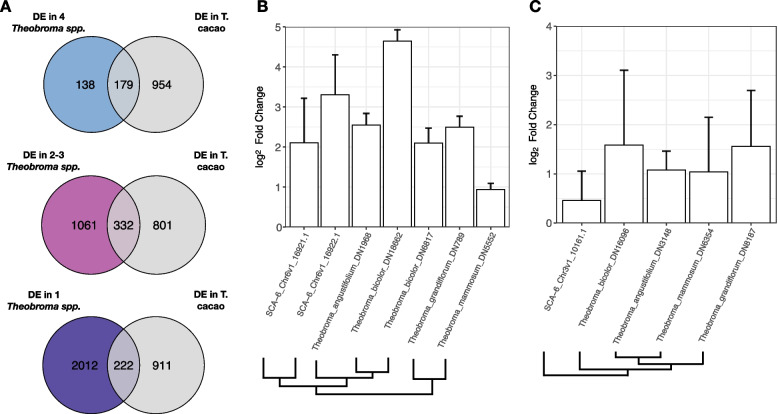
Transcriptome responses in wild *Theobroma* species reveal orthologous defenses. **A** Venn diagrams displaying overlap between genes that are differentially expressed in at least one population of *T. cacao* and supertranscripts that are differentially expressed in 4 (top), 2–3 (middle), or 1 (bottom) non-cacao *Theobroma* species. **B** Log_2_ fold changes (± SE) for genes and superstranscripts from closely related *Theobroma* species in orthogroup 60, berberine bridge enzymes. Cladogram represents gene family relationships. **C** Log_2_ fold changes (± SE) for genes and superstranscripts from closely related *Theobroma* species in orthogroup 361, WRKY transcription factors. Cladogram represents gene family relationships

To test this hypothesis, we investigated the transcriptional response to pathogen challenge in four non-cacao *Theobroma* species: *T. angustifolium*, *T. bicolor*, *T. grandiflorum*, and *T. mammosum*. Through RNA sequencing and molecular evolutionary analyses, we identified gene families that respond consistently to pathogen challenge across four species of *Theobroma*. Using the *T. cacao* differential expression results from the previous section, combined with those from the non-cacao *Theobroma* species, we defined differentially expressed orthogroups. An orthogroup only needed a single differentially expressed supertranscript or gene to be considered a differentially expressed orthogroup. In total, 733 orthogroups were differentially expressed in both *T. cacao* and non-cacao species (Fig. 6A). Of these, 179 were differentially expressed in at least one *T. cacao* population and all four non-cacao *Theobroma* species (hereafter referred to as core orthogroups). For most orthogroups, mean LFC was weakly, albeit significantly, correlated across *Theobroma spp.* and *T. cacao* (Additional file 2: Fig. S10). Several of these core orthogroups, however, had strong responses (|LFC|> 1) across both datasets (Additional file 1: Table S11). Thus, while LFC may not be strongly correlated in a broad sense, some orthogroups seemed to be consistently important for *Theobroma*’s defense response.

These consistently responding orthogroups included a diverse array of gene families with both well-known and potentially novel roles in defense (Additional file 1: Table S11). Similar to the cacao-only results, we observed both the chitinase and endochitinase gene families, proteins known to be antipathogenic in many species, including cacao [112]. Likewise, four gene families involved in the biosynthesis and modification of hydroxycinnamic acids were also observed. Isoeugenol synthases, a family of proteins responsible for the biosynthesis of the broad-spectrum antimicrobial phenolic isoeugenol, were upregulated 3–32 fold in each species (Additional file 1: Table S11) [130, 131].

Perhaps the two most interesting orthogroups, however, were OG60 and OG361, which contained berberine bridge and WRKY transcription factor proteins, respectively. Cacao genes in each of these families, *TcBBE8* (SCA6_Chr6v1_16921) and *TcWRKY29* (SCA6_Chr3v1_10161), were differentially expressed upon pathogen challenge and displayed signatures of diversification indicative of selection in resistant varieties (Figs. 3 and 5). Phylogenies for OG60 and OG361 revealed closely related orthologs that were responding consistently across species (Additional file 2: Figs. S11-S12). Supertranscripts (see “Methods”) belonging to the same clade as *TcBBE8* were 2–24 fold upregulated in response to pathogen challenge (Fig. 6B), while those in the same clade as *TcWRKY29* were 2–3 fold upregulated (Fig. 6C). Moreover, we also observed consistent upregulation of two other defense-associated WRKY transcription factors, *TcWRKY22* (SCA6_Chr1v1_03377), and *TcWRKY69* (SCA6_Chr6v1_18337) (Additional file 2: Figs. S11-S12). Such consistent responses across different species, time points (8hpi vs 48hpi), experimental designs, and pathogen strains, suggests these two gene families are likely key components of cacao’s defense response.

### Conserved orthogroups show evidence of positive selection

To examine how selection shaped the conserved aspects of *Theobroma*’s defense outlined above, we performed branch-site tests using BUSTED [100] to look for evidence of episodic diversifying selection. We compared core orthogroups with mean |LFC|> 1 to an equal number of orthogroups selected at random. Of the 48 core orthogroups with mean |LFC|> 1, 46 displayed significant (FDR-adjusted *p*-values < 0.05) signatures of positive selection (Additional file 2: Fig. S13), compared to just 31 for orthogroups selected at random. Thus, positive selection was significantly associated with orthogroup type (CORE vs random; chi-sq., *p* < 0.001; Additional file 2: Fig. S13). Together, these results suggest orthogroups that are consistently differentially expressed across *Theobroma spp.* following pathogen challenge are evolving under positive selection.

## Discussion

Plant pathogens are responsible for extensive annual yield loss in crop species, a problem that is likely to become worse due to climate change. Through breeding, humans have sought to mitigate the damage these pathogens cause by harnessing natural variation in resistance/susceptibility. However, hybrids created in plant breeding programs represent only a small proportion of a species’ overall genetic diversity. Wild populations of crop species are therefore important reservoirs of genetic diversity. Here, we used genomic, transcriptomic, and metabolomic data to investigate the evolution of defense response across four populations of cacao, with the goal of identifying novel resistance alleles that could potentially be incorporated into breeding programs.

Differential expression analysis revealed a rich set of defense-associated genes that change their expression level either in response to pathogen challenge or between resistant/susceptible individuals. Many of these differentially expressed genes (30–40%) are unique to each population (Fig. 2A). That is, ~40% of genes that were differentially expressed in one population were not differentially expressed in the other three. Despite this high degree of lineage specificity in transcriptional response, many DEGs appear to be involved in a common set of biological processes (Fig. 2C). These include both broad (e.g., induced systemic resistance) and specific (e.g., cinnamic acid biosynthetic process) categories. Furthermore, although 30–40% of the genes belonging to these shared GO terms were lineage-specific (Fig. 3C), many of them have a high potential for functional redundancy. For instance, within the cinnamic acid biosynthetic pathway, we observed lineage-specific expression and/or evolutionary rate differences in four genes encoding putative caffeic acid 3-O methyltransferases (*TcCOMT*), as well as two genes for both shikimate O-hyroxycinnamoyltransferase (*TcHST*) and laccase-14 (*TcLAC14*). Likewise, for the lignin biosynthetic pathway, we observed four putative *TcHST* genes and seven laccase genes. Thus, while each of our populations likely possess unique solutions to pathogen challenge, at least a portion of their defense responses seem to converge upon common pathways. Some of the variation may represent lineage-specific differences in the timing of defense gene regulation. It may also result from lineage-specific co-evolution with pathogen effectors, which could drive high evolutionary rates and divergence among genetically isolated host lineages.

Of the nine processes that were enriched across all four populations, either in response to pathogen challenge or R/S phenotype, lignin biosynthetic process and cinnamic acid biosynthesis stand out for several reasons. First, as part of the phenylpropanoid pathway, both processes are well-known contributors to plant defense against a wide range of pathogens. For instance, lignin and monolignols play a role in hypersensitive response and penetration defense against fungi and oomycetes [132, 133]. Genes involved in lignin biosynthesis interact with nucleotide-binding leucine-rich repeat proteins to modulate plant defense [123]. Hydroxycinnamic acid amides such as *p*-coumaroylagmatine, feruloylagmatine, *p*-coumaroylputrescine, and feruloylputrescine confer defense to the fungal pathogen *Alternaria brassicicola* in *Arabidopsis thaliana* [125]. The phenolic aldehyde vanillin, a derivative of ferulic acid, hinders the growth of multiple bacterial species by dissipating ion gradients and thereby inhibiting respiration [126]. The hydroxycinnamic acid amide clovamide indirectly inhibits the growth of three species of *Phytophthora*, including *P. palmivora*, in cacao [83]. And lastly, caffeic acid and its derivatives both directly and indirectly inhibit many pathogens, among them *P. palmivora* and *P. megakarya* [86, 127].

The last of these compounds, caffeic acid, is particularly interesting because the gene responsible for catalyzing the reaction from caffeoyl shikimate to caffeic acid, *TcCSE*, displays consistent upregulation across all four populations (Fig. 4A). To test whether caffeic acid and *TcCSE* were involved in defense response against *P. palmivora*, we performed a series of experiments. We first verified the function of *TcCSE* through heterologous overexpression in *N. benthamiana*, confirming the accumulation of caffeic acid both 48 and 96 h post transformation (Fig. 4B). Caffeic acid was inhibitory to *P. palmivora* mycelia (Fig. 4C). Despite these results, however, genotypes displaying upregulated *TcCSE* in our transcriptome experiment did not display increased caffeic acid accumulation in cacao leaves transiently expressing the gene (Fig. 4E). This result could be due to multiple factors. First, *TcCSE* expression could precede caffeic acid accumulation. The fact that the *TcCSE* overexpression experiment (Fig. 4B) was collected 8 h post inoculation may not have provided sufficient time for metabolite accumulation. Second, it could be the case that caffeic acid was converted into lignin via sinapic acid [134], which would not be detected using our metabolite extraction protocol. And lastly, caffeic acid could have been converted into one or more caffeic acid derivatives that are difficult to predict and quantify [127]. Together, our results indicate that *TcCSE* and caffeic acid are potentially important components of the cacao plant defense, though we so far lack a complete understanding of expression time course and the fate of resulting metabolites. Additionally, accumulation of theobromine (Fig. 4D) in the leaf extracellular space in response to zoospore inoculation might be a first line of defense, considering its reported antimicrobial activity [129], whereas caffeic acid derivatives may provide protection internally after hyphae penetration.

We found that major aspects of cacao plants defense responses against *P. palmivora* were lineage-specific, and, therefore, resistance appears to be mediated by different genes depending on the population. To further test this possibility, we estimated lineage-specific adaptation associated with each population’s resistant genotypes. Similar to our differential expression results, there was no consistent set of rapidly evolving resistance-associated genes across all four populations. That is, different sets of genes displayed evidence of selection in each population’s resistant genotypes (Fig. 5B). Three of the genes displaying evidence of selection are also differentially expressed upon pathogen exposure in all four populations: *TcWRKY29* (SCA6_Chr3v1_10161), *TcBBE8* (SCA6_Chr6v1_16921), and *TcFMO1* (SCA6_Chr9v1_23321) (Fig. 5C; Additional file 2: Figs. S11-S12). Two of these genes, *TcBBE8 and TcWRKY29*, were also differentially expressed in non-cacao *Theobroma* species inoculated with *P. palmivora* and belong to orthogroups that display signatures of positive selection (Fig. 6). While a large portion of cacao’s defense response appears to be lineage-specific, consistent transcriptional responses and signatures of adaptation among a small set of orthologous genes suggest certain components of cacao’s defense predate its speciation. Despite multiple lines of evidence supporting the importance of these genes, none of them appear to be present in predicted resistance QTLs [29, 31]. This observation has two likely explanations. First, QTLs are often predicted based on progeny from only a handful of parent clones that represent a small fraction of cacao’s overall genetic diversity. By broadening the search for candidate genes to a more diverse set of germplasm, we are able to capture new, previously unidentified genes. Therefore, while differences among cacao populations represent novel opportunities for breeding, conserved genes that respond consistently across diverse genotypes but have not always been detected, similarly represent valuable breeding targets. The second possibility, however, has to do with experimental design. *P. palmivora* primarily infects cacao pods. And, while numerous publications show pod inoculation and leaf inoculation yield highly correlated phenotypes [135–138], it is possible that genes identified using the former methodology would not necessarily match genes identified using the latter.

## Conclusions

Producing cacao varieties that are durably resistant to pathogens requires the development of crop improvement methods that harness underutilized germplasm and rapidly identify alleles associated with disease resistance. With high-throughput sequencing and readily available analytical tools, we are now in an era where the benefits of genetic diversity in cacao and other long generation time plants can be more fully realized. In this study, we investigated the evolution of defense response against *P. palmivora* across four divergent populations of cacao. Consistent with the high genetic differentiation among these populations, we observed both population-specific transcriptional differences and historical responses to selection indicating that these populations have adapted to their local microbial communities in ways that affect their defenses against *P. palmivora*. Genes and pathways that responded consistently across all four populations include *TcCSE*, *TcFMO1*, *TcWRKY29*, and *TcBBE8*, as well as pathways involved in the biosynthesis of phenylpropanoids (Additional file 2: Figs. S11-S12). Together, our results indicate cacao’s defenses against *P. palmivora* are mediated by a network of both conserved and diverged responses, and suggest wild populations are a source of genetic diversity that could help improve both the health and resilience of cacao.

### Supplementary Information

**Additional file 1: Table S1.** Summary of sequence data for Theobroma cacao genotypes used in the draft assemblies for Theobroma cacao genotypes. **Table S2.** The length-weighted mean molecule length for Supernova genotype assemblies at different read depths estimated from linked reads. **Table S3.** Estimated k-mer based genome characteristics for Theobroma cacao genotypes **Table S4.** BUSCO completeness scores for each cacao meta-assembly. **Table S5.** Genome summary statistics for the T. cacao reference genomes, Matina and Criollo, and new meta-assemblies. **Table S6.** RNA samples pooled for genome annotation. **Table S7.** Primers used to clone TcCSE (SCA-6_Chr6v1_17513) and their cut sites. **Table S8.** Annotated MS2 fragmentation patterns from LC-MS/MS data collected during zoospore infection. **Table S9.** Genes in the top 1% of their respective PBS distributions that also overlap a differentially expressed gene. **Table S10.** Genes in the top 1% of their respective population branch statistic (PBS) distributions. **Table S11.** Orthogroups that are differentially expressed in response to P. palmivora challenge in both wild Theobroma spp. and Theobroma cacao. **Table S12.** Meta-data for the RNA-seq experiment. **Table S13.** Top 1000 genes from each genetic group in response to pathogen treatment, ranked by absolute log fold change (|LFC|). **Table S14.** Top 1000 genes from each genetic group in response tolerance/susceptibility phenotype, ranked by absolute log fold change (|LFC|). **Table S15.** Meta-data for metabolite extraction from the RNA-seq experimental tissues.

**Additional file 2: Fig. S1.** Distribution of biological replicates for each genotype included in the transcriptome experiment. **Fig. S2.** Experimental design. **Fig. S3.** Environmental covariates included in the GLM used for differential expression. **Fig. S4.** Expression of differentially expressed genes that are either unique to a single population or shared across populations, for P. palmivora treatment or R/S phenotype. **Fig. S5.** Proportion of genes that are unique to each population for various sized subsamples, ranging from 200 to 2000 genes, for P. palmivora treatment or R/S phenotype. **Fig. S6.** Overlap of differentially expressed closely related paralogs (i.e. paralogous genes with ≥ 95% identity). **Fig. S7.** Overlap of differentially expressed orthogroups (i.e. orthogroups containing 1 or more differentially expressed genes). **Fig. S8.** Pairwise spearman correlations of mean log2 fold changes for all orthogroups included in this study. **Fig. S9.** Pairwise spearman correlations of log2 fold changes for 1:1 orthologs between A. thaliana and its close relatives and between accessions of A. thaliana. **Fig. S10.** Differentially expressed orthogroups in T. cacao and non-cacao Theobroma spp. **Fig. S11.** Maximum-likelihood gene family phylogeny for orthogroup 60, FAD-binding berberine bridge enzymes. **Fig. S12.** Maximum-likelihood gene family phylogeny for orthogroup 361, WRKY transcription factors. **Fig. S13.** Orthogroups with signatures of positive selection.

## Data Availability

Raw sequence data, for both the whole-genome sequencing and RNA-sequencing experiments, are deposited to the National Center for Biotechnology Information Sequence Read Archive under the BioProject PRJNA558793, and can be found at http://identifiers.org/bioproject:PRJNA558793. Genome assemblies can be found under GenBank accessions CP139290-CP139299. Count matrices can be found at the following link: http://bigdata.bx.psu.edu/Cacao_NSF_data/assemblies/. Custom scripts for analyses and plots can be found on GitHub at the following link: https://github.com/npwinters/NSF_Cacao_RNAseq_MS. RNA-seq data from the Winkelmüller et al. *Arabidopsis* flg22 experiments can be found in NCBI’s Gene Expression Omnibus under the accession GSE115991 [107].

## Abbreviations

QTL: Quantitative trait loci
SNP: Single-nucleotide polymorphism
CATIE: Tropical Agricultural Research and Higher Education Center, Turrialba, Costa Rica
IC3: International Cocoa Collection
NCBI: National Center for Biotechnology Information
*nt*: NCBI nucleotide collection database
*nr*: NCBI non-redundant protein database
BUSCO: Benchmarking universal single-copy orthologs
BLAST: Basic local alignment search tool
LFC: Log fold change (in this manuscript, log_2_)
GO: Gene ontology
DAG: Directed acyclic graph
CSE: Caffeoyl shikimate esterase
UPLC: Ultra-performance liquid chromatography
LC-MS/MS: Liquid chromatography with tandem mass spectrometry
PBS: Population branch statistic
HMM: Hidden Markov model
BUSTED: Branch-site unrestricted statistical test for episodic diversification
DEG: Differentially expressed gene
GH3: Gretchen Hagen 3
BBE8: Berberine bridge enzyme 8
NLR: Nucleotide-binding leucine-rich repeat proteins
LRK: Leucine-rich repeat serine/threonine protein kinase
MATE: Multidrug and toxin extrusion
HCAA: Hydroxycinnamic acid amide
OG: Orthologous group (orthogroup)
COMT: Caffeic acid 3-O methyltransferase
HST: Shikimate O-hydroxycinnamoyltransferase
LAC15: Laccase 14
FMO1: Flavin containing dimethylaniline monoxygenase 1
