# Supplementary material for: A combination of conserved and diverged responses underlies Theobroma cacao’s defense response to Phytophthora palmivora
Source: BMC Biol. 2024 Feb 16;22:38. doi: 10.1186/s12915-024-01831-2 (PMC10870529; doi:10.1186/s12915-024-01831-2)
Supplement: Supplementary file 2 — Additional file 2: Fig. S1. Distribution of biological replicates for each genotype included in the transcriptome experiment. Fig. S2. Experimental design. Fig. S3. Environmental covariates included in the GLM used for differential expression. Fig. S4. Expression of differentially expressed genes that are either unique to a single population or shared across populations, for P. palmivora treatment or R/S phenotype. Fig. S5. Proportion of genes that are unique to each population for various sized subsamples, ranging from 200 to 2000 genes, for P. palmivora treatment or R/S phenotype. Fig. S6. Overlap of differentially expressed closely related paralogs (i.e. paralogous genes with ≥ 95% identity). Fig. S7. Overlap of differentially expressed orthogroups (i.e. orthogroups containing 1 or more differentially expressed genes). Fig. S8. Pairwise spearman correlations of mean log2 fold changes for all orthogroups included in this study. Fig. S9. Pairwise spearman correlations of log2 fold changes for 1:1 orthologs between A. thaliana and its close relatives and between accessions of A. thaliana. Fig. S10. Differentially expressed orthogroups in T. cacao and non-cacao Theobroma spp. Fig. S11. Maximum-likelihood gene family phylogeny for orthogroup 60, FAD-binding berberine bridge enzymes. Fig. S12. Maximum-likelihood gene family phylogeny for orthogroup 361, WRKY transcription factors. Fig. S13. Orthogroups with signatures of positive selection. [file 12915_2024_1831_MOESM2_ESM.pdf]

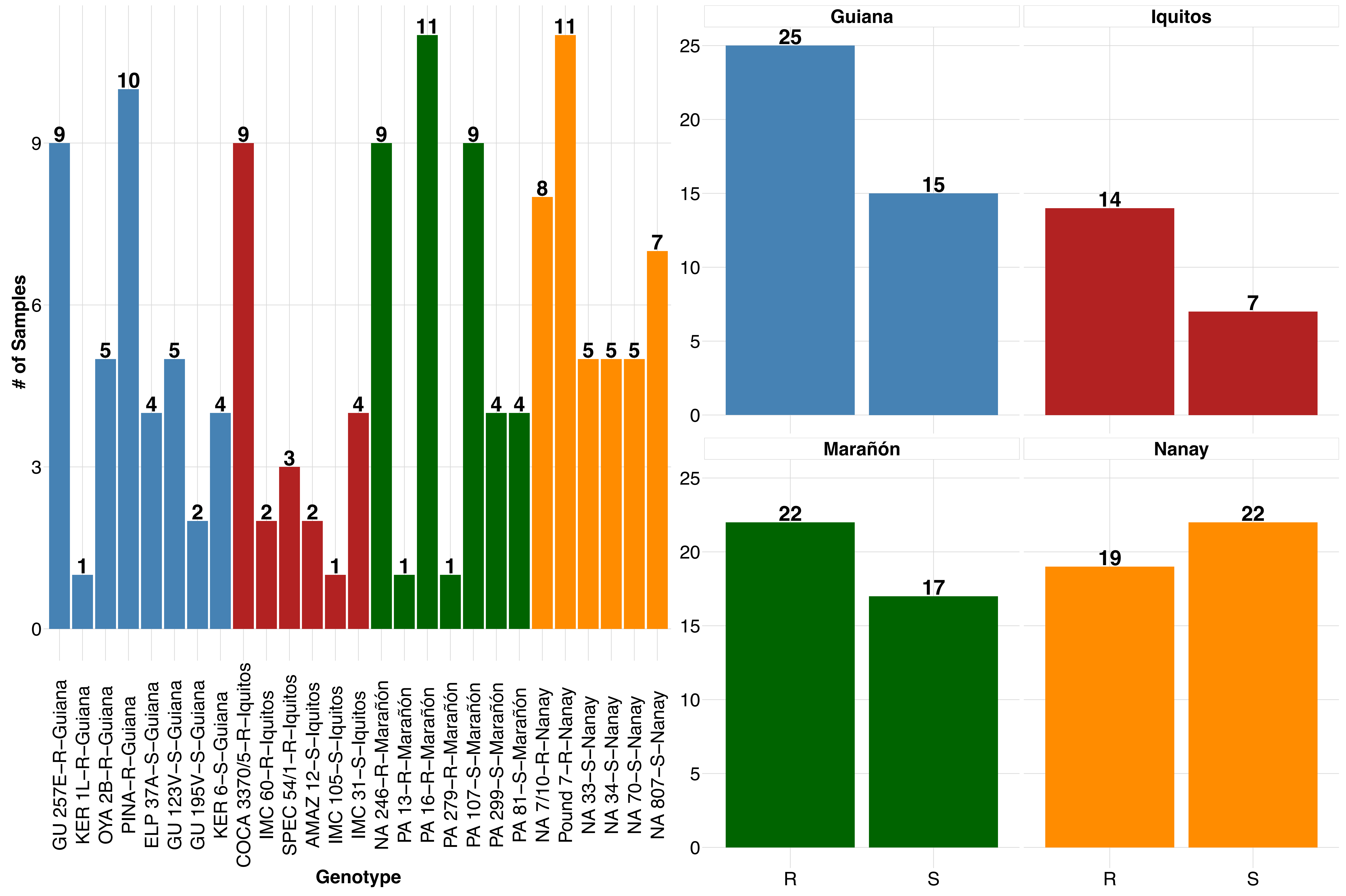

Supplemental Figure S1. Distribution of biological replicates for each genotype included in the transcriptome experiment. Color indicates population membership: Guiana (blue), Iquitos (red), Marañón (green), and Nanay (orange). (R) indicates resistant genotypes and (S) indicates susceptible genotypes.

Bench 1

Tray A (P. pal)

	1	2	3	4	5
1	NA 34	SCA 6	NA 807	NA 33	NA 7/10
2	KER 6	NA 807	COCA 3370/5	NA 246	GU 123V
3	PA 16	ICS 1	NA 7/10		ICS 1
4	GU 257E	AMAZ 12	GU 257E	PA 299	PA 107
5	PA 81		PA 16	COCA 3370/5	ICS 1
6	PINA	GU 257E	Pound 7	KER 1L	NA 246

Tray B (water)

	1	2	3	4	5
1			PA 107	COCA 3370/5	GU 195V
2	OYA 2B	SCA 6	NA 70	COCA 3370/5	
3	NA 246	NA 70	PA 16	PINA	PA 16
4	ICS 1	PA 299	IMC 105	Pound 7	NA 7/10
5	ELP 37A	Pound 7	NA 246	GU 257E	IMC 31
6	NA 807	NA 7/10	NA 807	PINA	IMC 60

Tray C (P. pal)

	1	2	3	4	5
1	PA 16	ICS 1	NA 70	PINA	PA 107
2	NA 807	GU 257E	Pound 7	PA 107	NA 246
3		OYA 2B	SPEC 54/1	KER 6	SCA 6
4	PINA	PA 13	COCA 3370/5	Pound 7	SCA 6
5	PA 81		NA 34	COCA 3370/5	IMC 31
6	NA 34	GU 123V	NA 33	ELP 37A	

Bench 2

Tray A (water)

	1	2	3	4	5
1	NA 7/10	NA 33	NA 807	SCA 6	NA 34
2	GU 123V	NA 246	COCA 3370/5	NA 33	KER 6
3	ICS 1	SPEC 54/1	NA 7/10	ICS 1	PA 16
4	PA 107	PA 299	OYA 2B	AMAZ 12	GU 257E
5	ICS 1	COCA 3370/5	PA 16		PA 81
6	NA 246	GU 195V	Pound 7	GU 257E	PINA

Tray B (P. pal)

	1	2	3	4	5
1	GU 123V	COCA 3370/5	PA 107		
2		COCA 3370/5	Pound 7	SCA 6	OYA 2B
3	PA 16	PINA	PA 16	NA 70	PA 279
4	NA 7/10	Pound 7	PA 107	PA 299	ICS 1
5	IMC 31	GU 257E	NA 246	Pound 7	ELP 37A
6	IMC 60	PINA	NA 807	NA 7/10	NA 807

Tray C (water)

	1	2	3	4	5
1	PA 107	PINA	NA 70	ICS 1	PA 16
2	NA 246	PA 107	Pound 7	GU 257E	NA 807
3	SCA 6	KER 6	SPEC 54/1	OYA 2B	
4	SCA 6	Pound 7	COCA 3370/5	PA 16	PINA
5	IMC 31	COCA 3370/5	ICS 1		PA 81
6		ELP 37A	NA 33	GU 123V	NA 34

Guiana resistant	Maranon resistant
Guiana susceptible	Maranon susceptible
Iquitos resistant	Nanay resistant
Iquitos susceptible	Nanay susceptible

Supplemental Figure S2. Two tables were aligned parallel to one another. On each bench, there were 3 trays, with approx. 30 plants on each tray. To minimize the effect of gradients in temperature, humidity, and light within the greenhouse, we kept the distance between tables to < 2 ft. We treated the plants in each tray with either pathogen or V8 control, such that parallel trays never experienced the same treatment. We randomized the placement of plants in each tray, with the caveat that the same genotype was in a mirrored position on both tables. Thus for each pair of plants within a genotype, one would receive pathogen treatment and one would receive control treatment. If there was an odd number of plants for a given genotype, or if a genotype only had one representative plant, the odd-numbered individual would be paired with an individual within the same population and resistance/susceptibility class. Lastly, if a genotype within the same population and resistance/ susceptibility class was unavailable, we used a genotype in the same resistance/susceptibility class from a different population. Color indicates population membership. ICS 1 and SCA 6 (purple) were included in the experiment but were not analyzed as part of this manuscript.

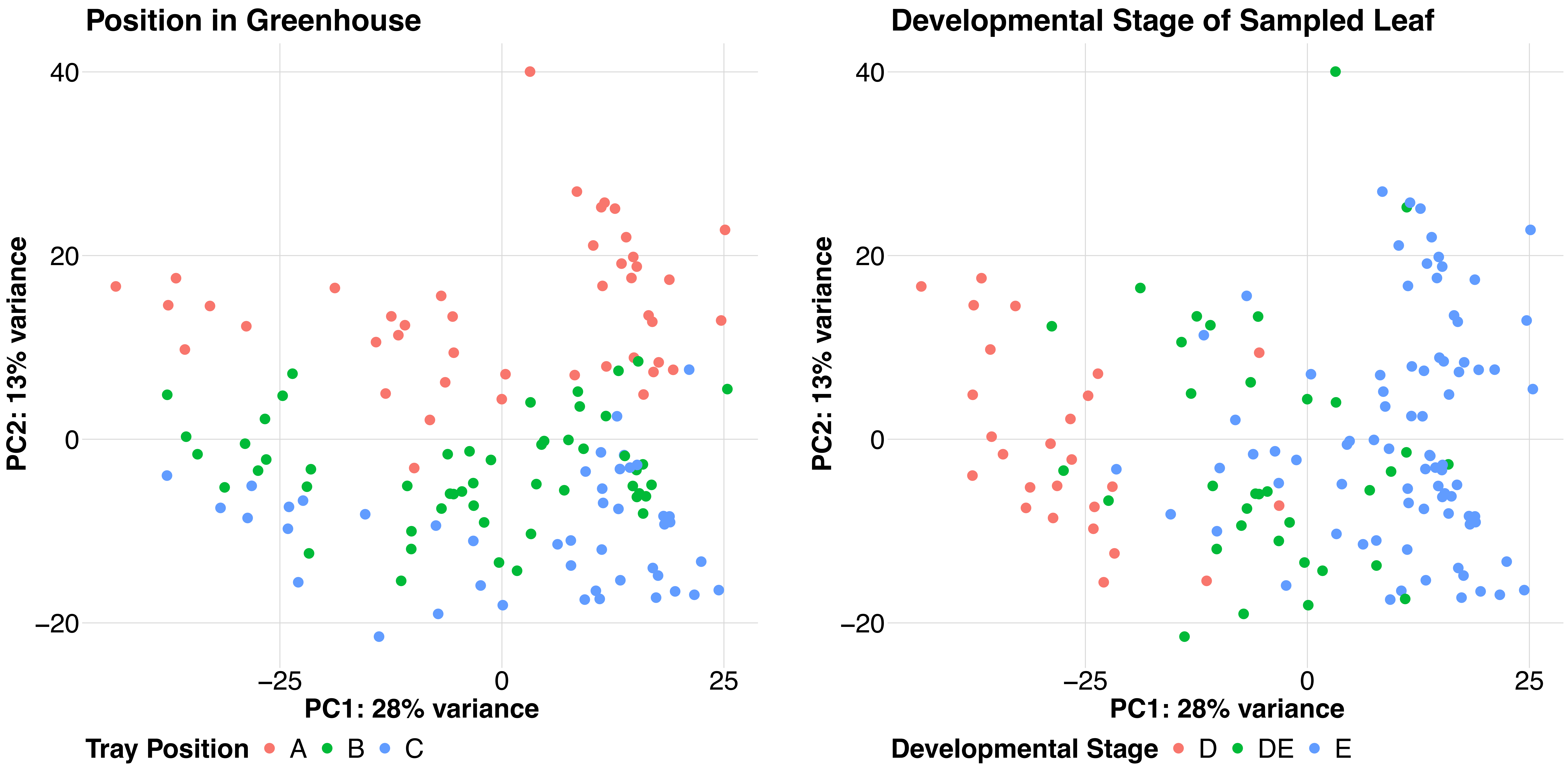

Supplemental Figure S3. Environmental covariates included in the GLM used for differential expression. (Left) tray position for each plant in the greenhouse, corresponding to supplemental figure S8. (Right) developmental stage of the leaves sampled for the transcriptome experiment.

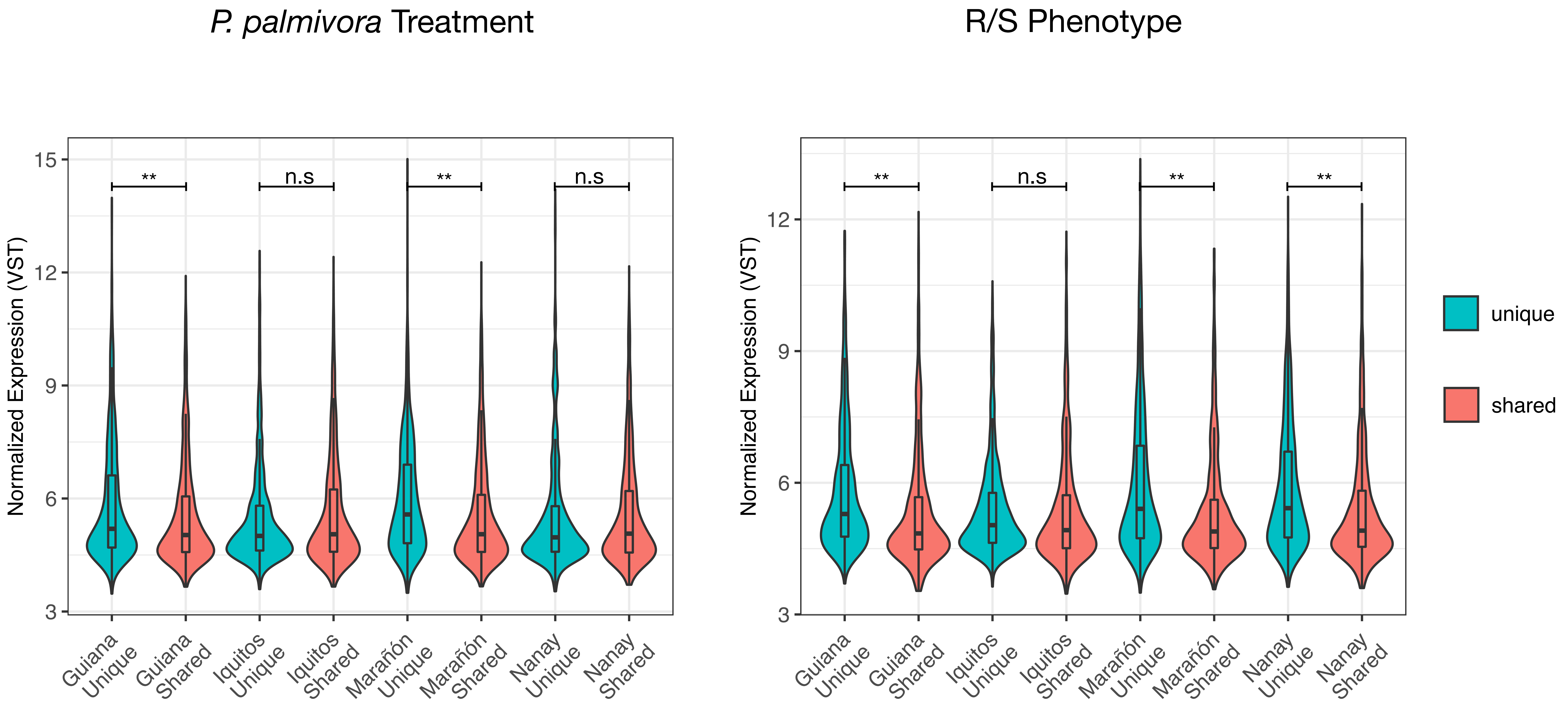

Supplemental Figure S4. Expression of differentially expressed genes that are either unique to a single population (blue) or shared across populations (red), for *P. palmivora* treatment (left) or R/S phenotype (right). Asterisks indicate statistical significance. For treatment, the genes unique to Guiana and Marañón had significantly higher expression than the genes shared among populations (one-way ANOVA, p -value < 2e-16; Tukey's HSD, FDR-adjusted p-value < 0.001). And for phenotype, the genes unique to Guiana, Marañón, and Nanay had significantly higher expression (one-way ANOVA, p -value < 2e-16; Tukey's HSD, FDR-adjusted p-value < 0.001).

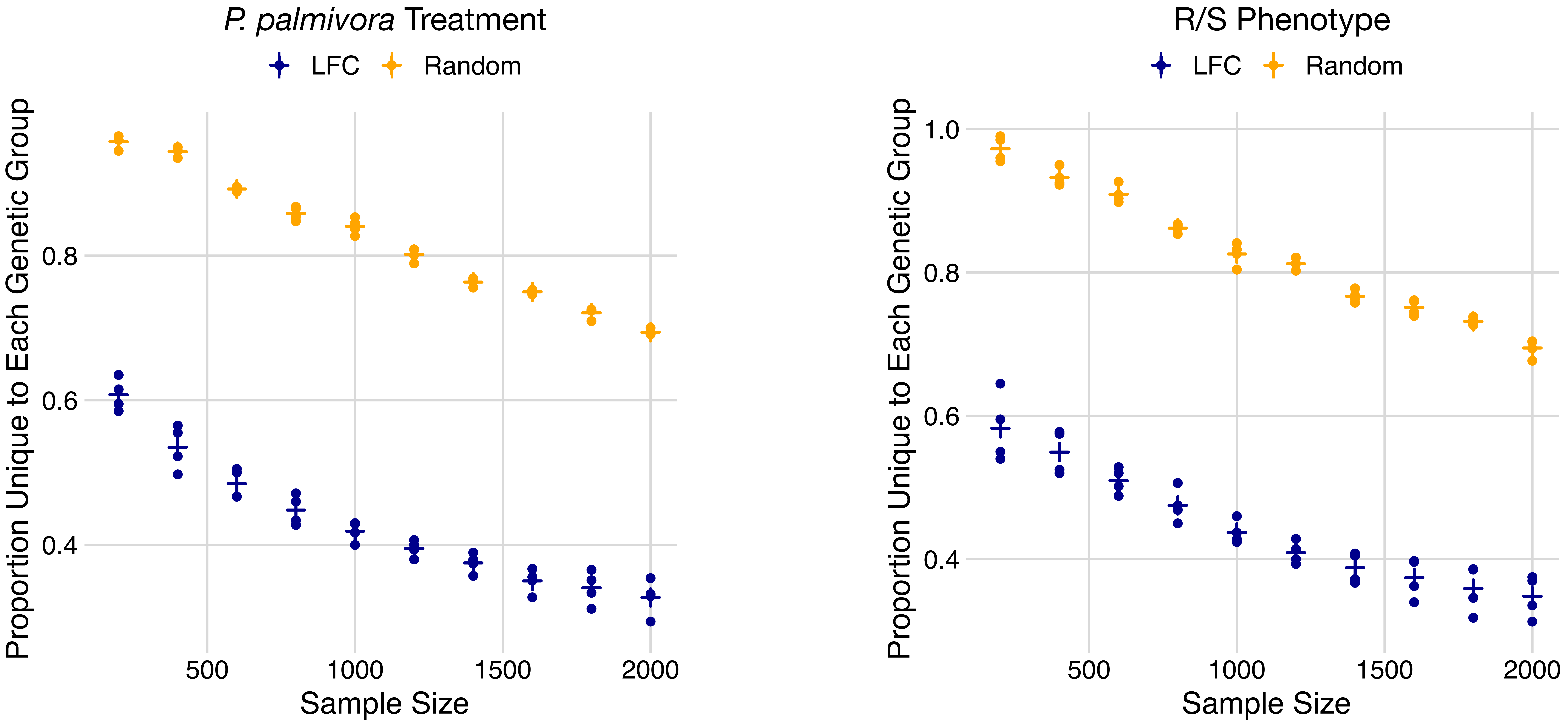

Supplemental Figure S5. Proportion of genes that are unique to each population for various sized subsamples, ranging from 200 to 2000 genes, for *P. palmivora* treatment (left) or R/S phenotype (right). Genes were either ranked by $|\log_2$ fold change$|$ before subsampling (blue), or subsampled at random (orange). Each dot represents one of four populations sampled. Means are represented as crosses. For every sample size, the proportion of genes unique to each population was significantly higher when the genes were drawn at random (one-way ANOVA, Proportion Unique Genes ~ Sample Size + Subsample Method + Sample Size:Subsample Method: p-values < 0.001).

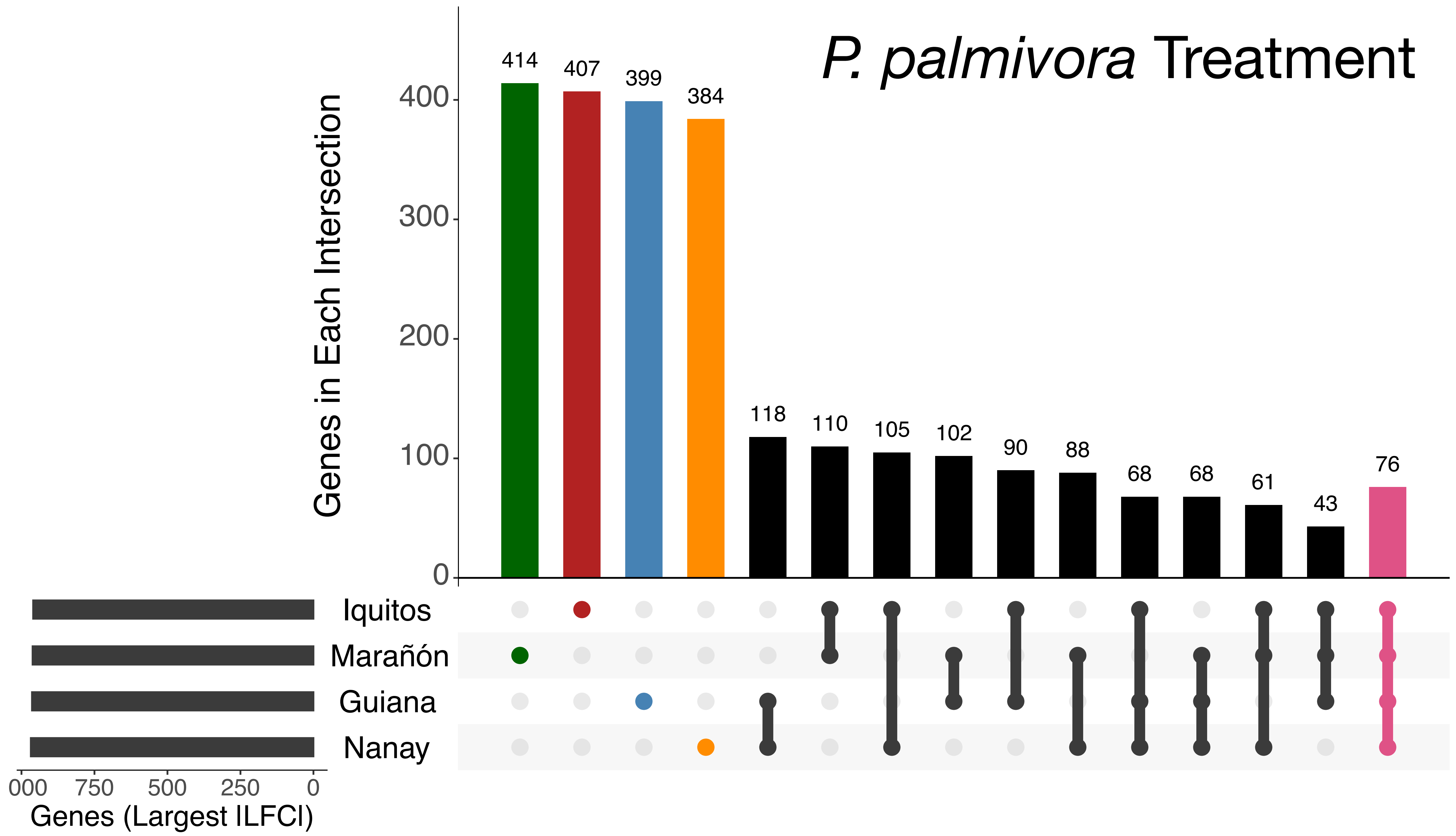

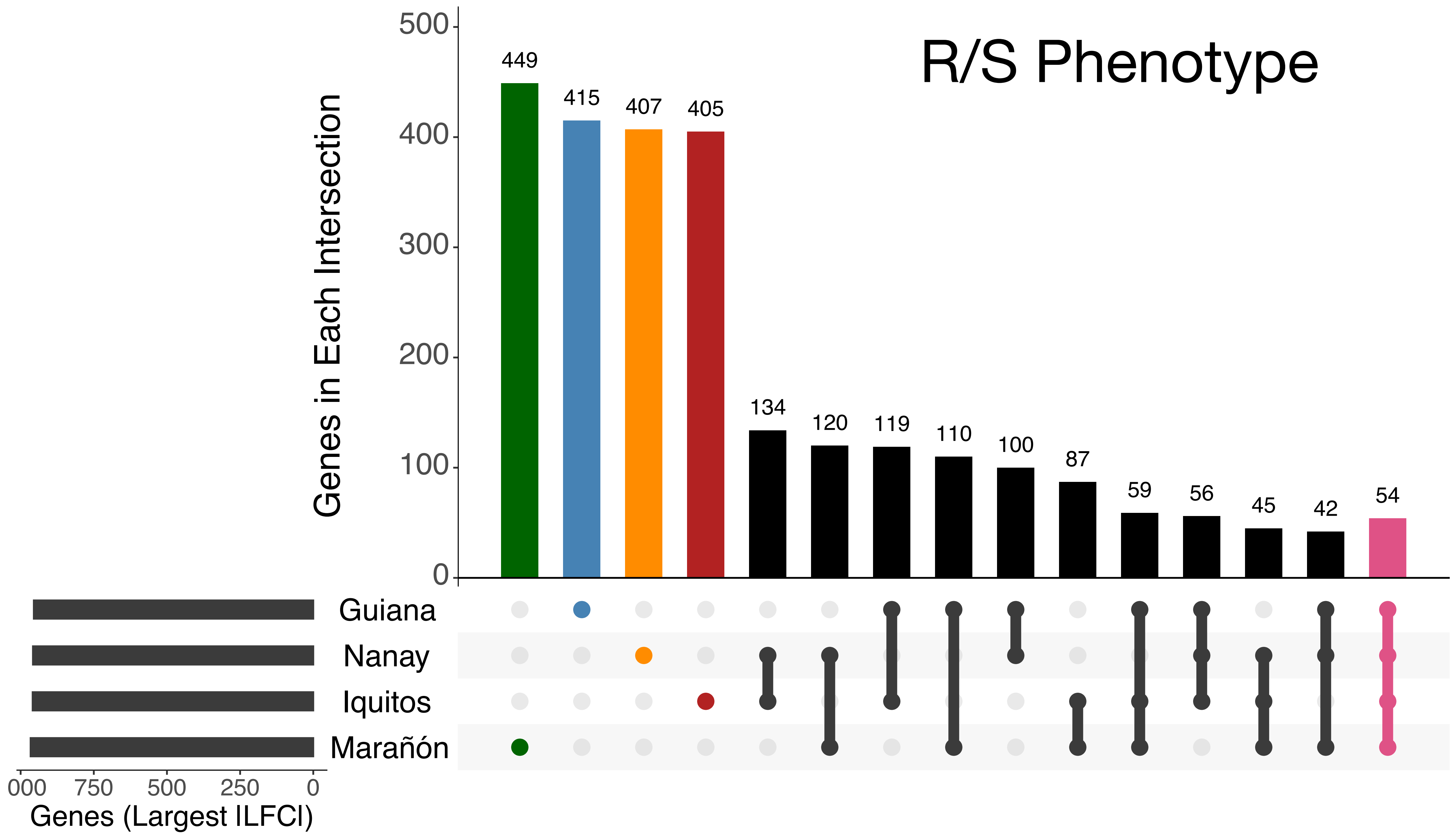

Supplemental Figure S6. Overlap of differentially expressed closely related paralogs (i.e. paralogous genes with ≥ 95% identity).The blue, red, green, and orange bars represent genes that are only DE in Guiana Iquitos, Marañón, or Nanay, respectively. The pink bar indicates paralogs that are DE across all four populations. Numbers above the bars indicate the number of paralogs in that specific intersection.

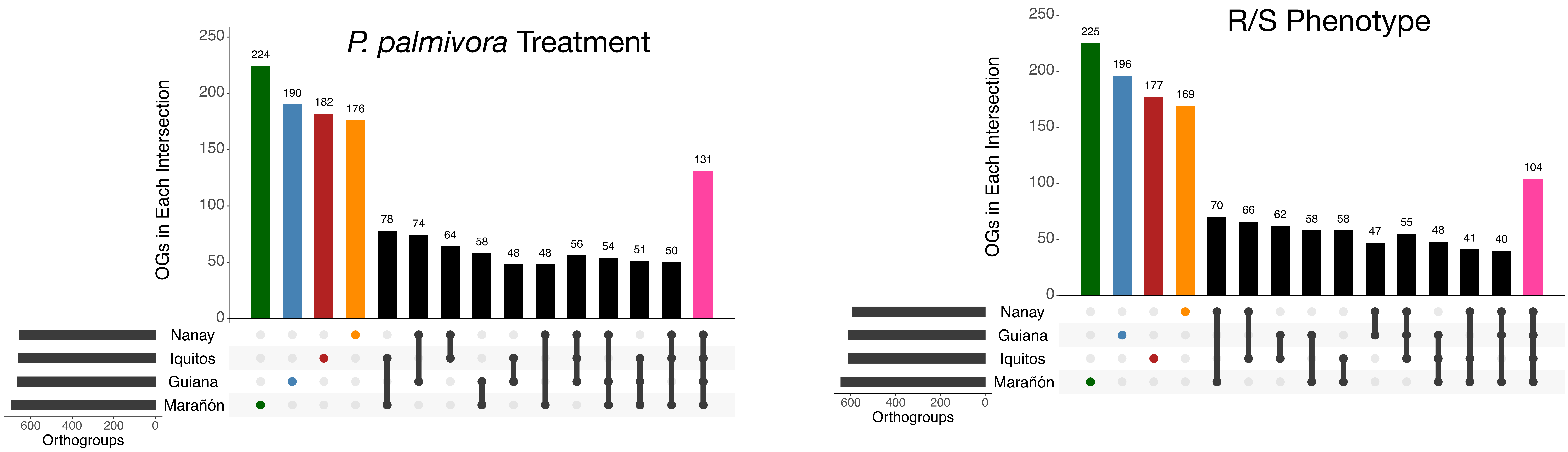

Supplemental Figure S7. Overlap of differentially expressed orthogroups (i.e. orthogroups containing 1 or more differentially expressed genes).The blue, red, green, and orange bars represent orthogroups that are only DE in Guiana Iquitos, Marañón, or Nanay, respectively. The pink bar indicates orthogroups that are DE across all four populations. Numbers above the bars indicate the number of orthogroups in that specific intersection.

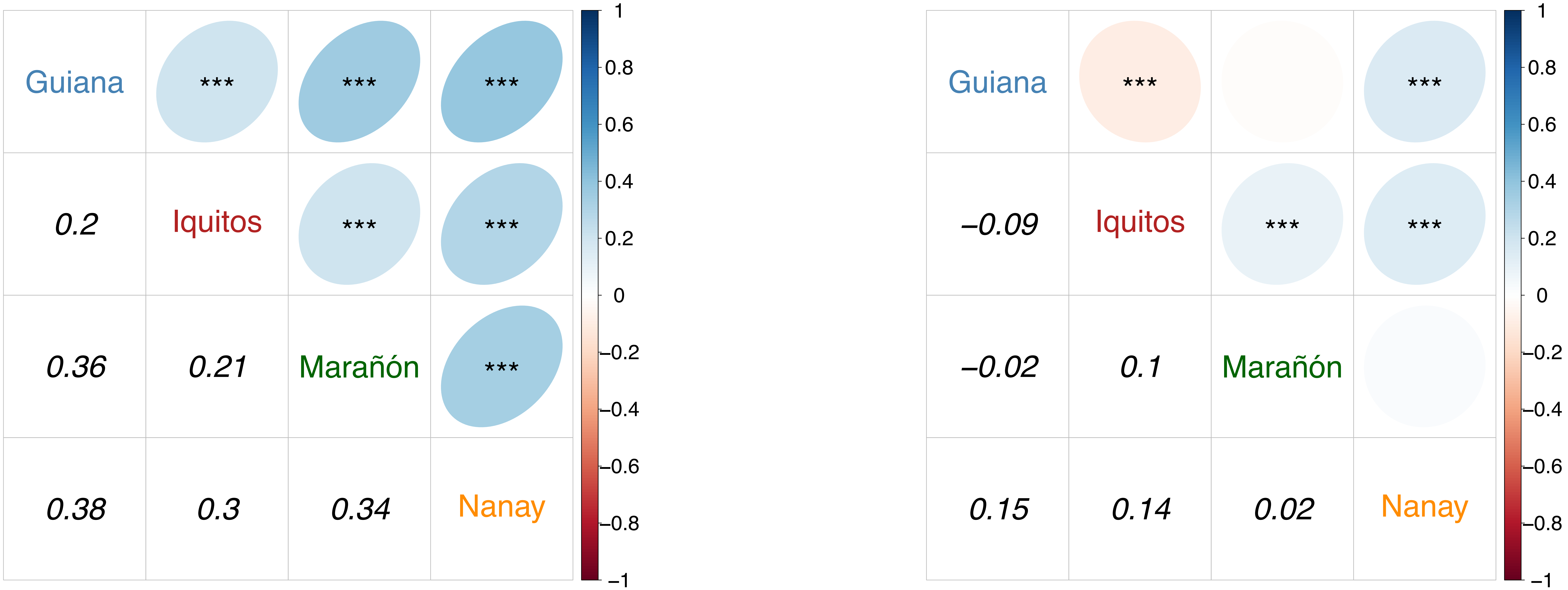

Supplemental Figure S8. Pairwise spearman correlations of mean log_2 fold changes for all orthogroups included in this study. All genes were first classified into orthogroups, then mean log_2 fold change for each orthogroup and population were then calculated. The bottom triangle is the Spearman correlation coefficient. The top triangle is the correlation coefficient depicted as an ellipse, the shape of which depends on the size of the coefficient. Stars indicate statistical significance ($p < 0.001$), tested using Spearman's rho.

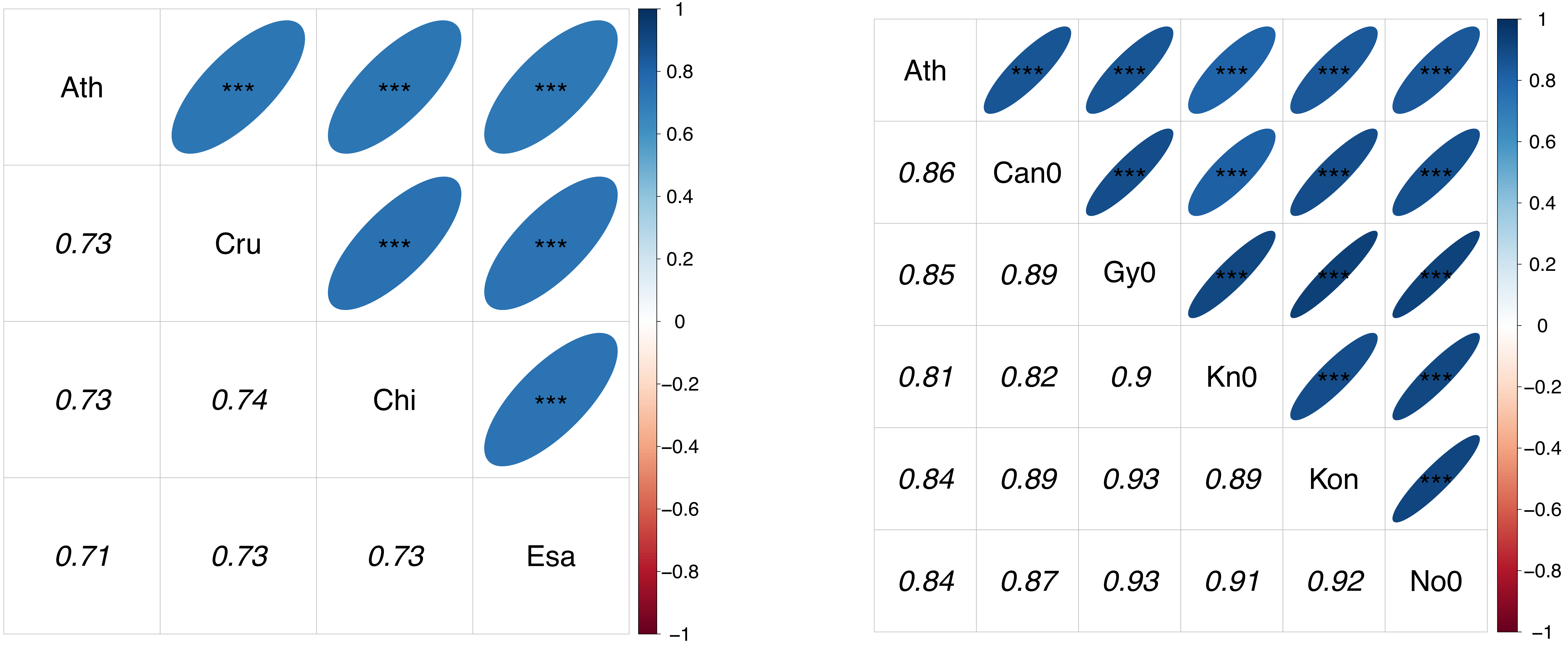

Supplemental Figure S9. Pairwise spearman correlations of $\log_2$ fold changes for 1:1 orthologs between *A. thaliana* and its close relatives (left) and between accessions of *A. thaliana* (right). The bottom triangle is the Spearman correlation coefficient. The top triangle is the correlation coefficient depicted as an ellipse, the shape of which depends on the size of the coefficient. Stars indicate statistical significance ($p < 0.001$), tested using Spearman's rho. Data are from Winkelmüller et al., 2021, *The Plant Cell*, Volume 33, Issue 6, June 2021, Pages 1863–1887.

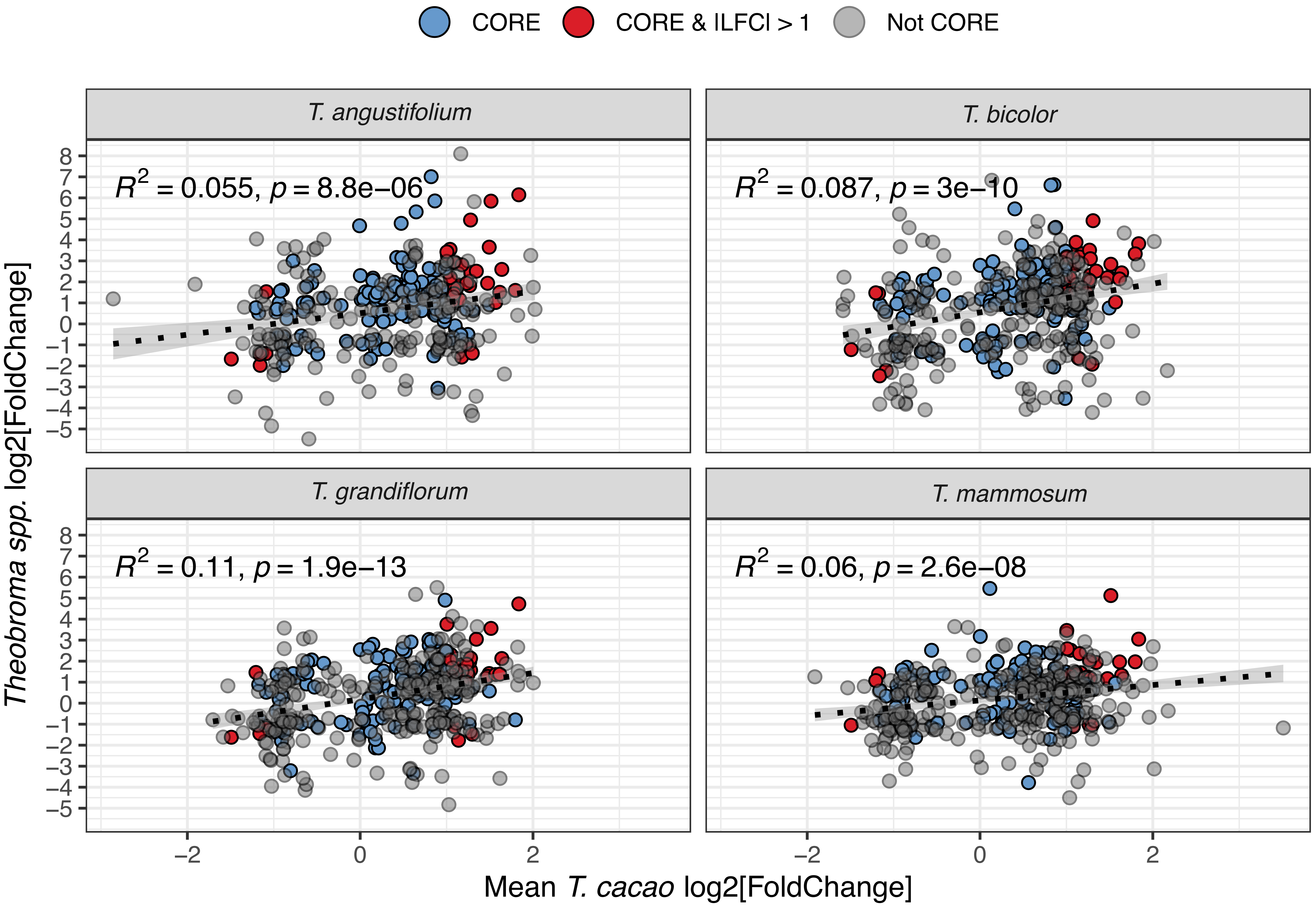

Supplemental Figure S10. Differentially expressed orthogroups in *T. cacao* and non-cacao *Theobroma spp.* Mean $\log_2$ fold change correlations between orthogroups differentially expressed in both *T. cacao* and each non-cacao *Theobroma spp.* Each point represents the mean $\log_2$ fold change for a single orthogroup. Blue points are CORE orthogroups whose mean |LFC| < 1 in *T. cacao,* non-cacao *Theobroma spp.*, or both. Red points are CORE orthogroups whose mean |LFC| > 1 in both *T. cacao* and non-cacao *Theobroma spp.* Gray points are not in the CORE resistance class.

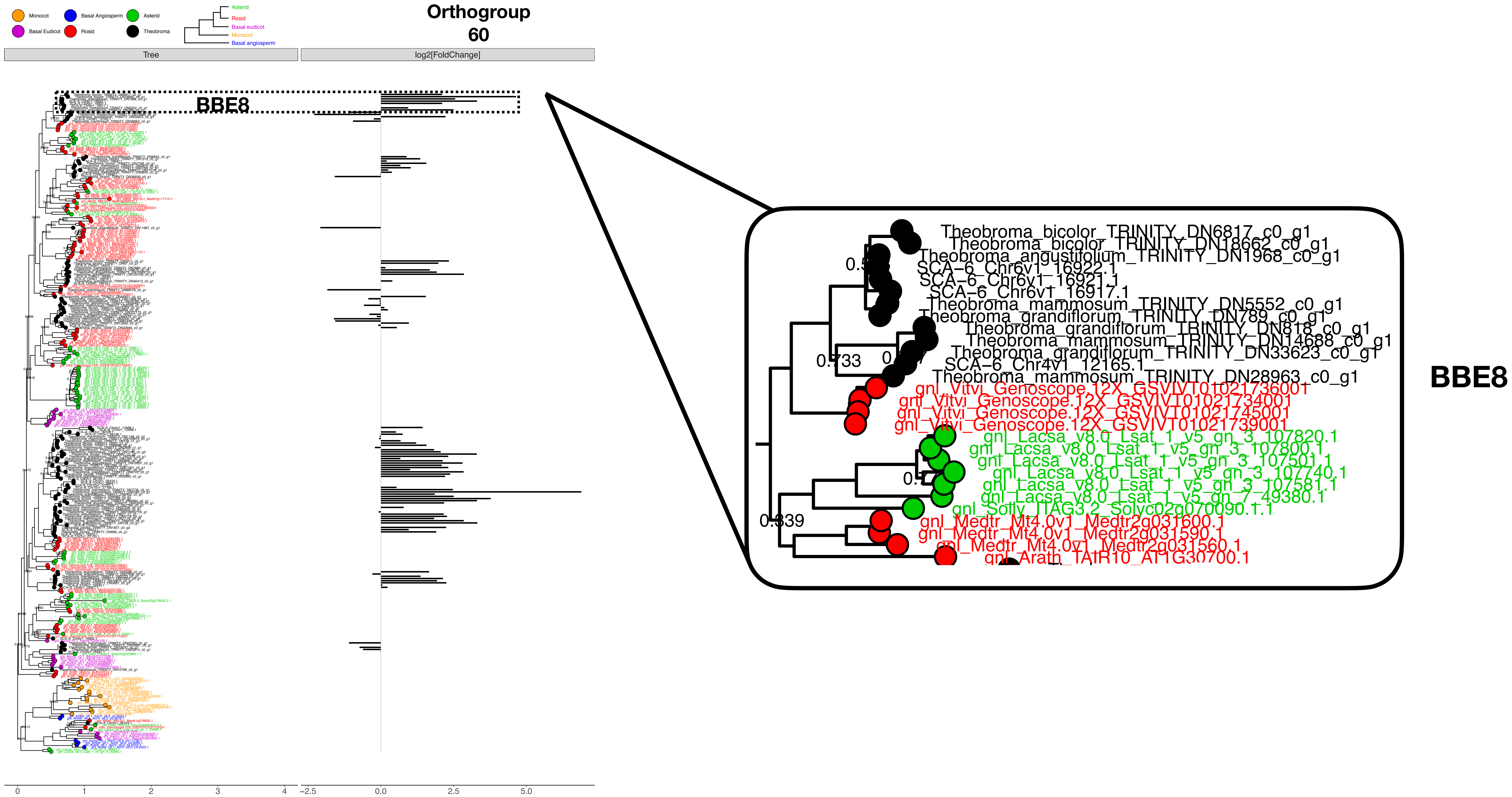

Supplemental Figure S11. Maximum-likelihood gene family phylogeny for orthogroup 60, FAD-binding berberine bridge enzymes. Sequence IDs are colored according to their lineage: basal angiosperm (blue), basal eudicot (purple), monocot (orange), rosid (red), and asterid (green). All *Theobroma* species, including *T. cacao*, are shown in black. *T. cacao* sequences are from the SCA-6 genome. Node values indicate SH-like local supports calculated by FastTree. SH supports > 80 are not shown. Bars in the right panel indicate $\log_2$ fold changes. The box indicates the clade containing TcBBE8 (SCA-6_Chr6v1_16921) and its close orthologs across *Theobroma.* Codon alignments were created using MAFFT v7.205 (L-INS-i) and trees were inferred using FastTree v2.1.10 (-nt -gtr),

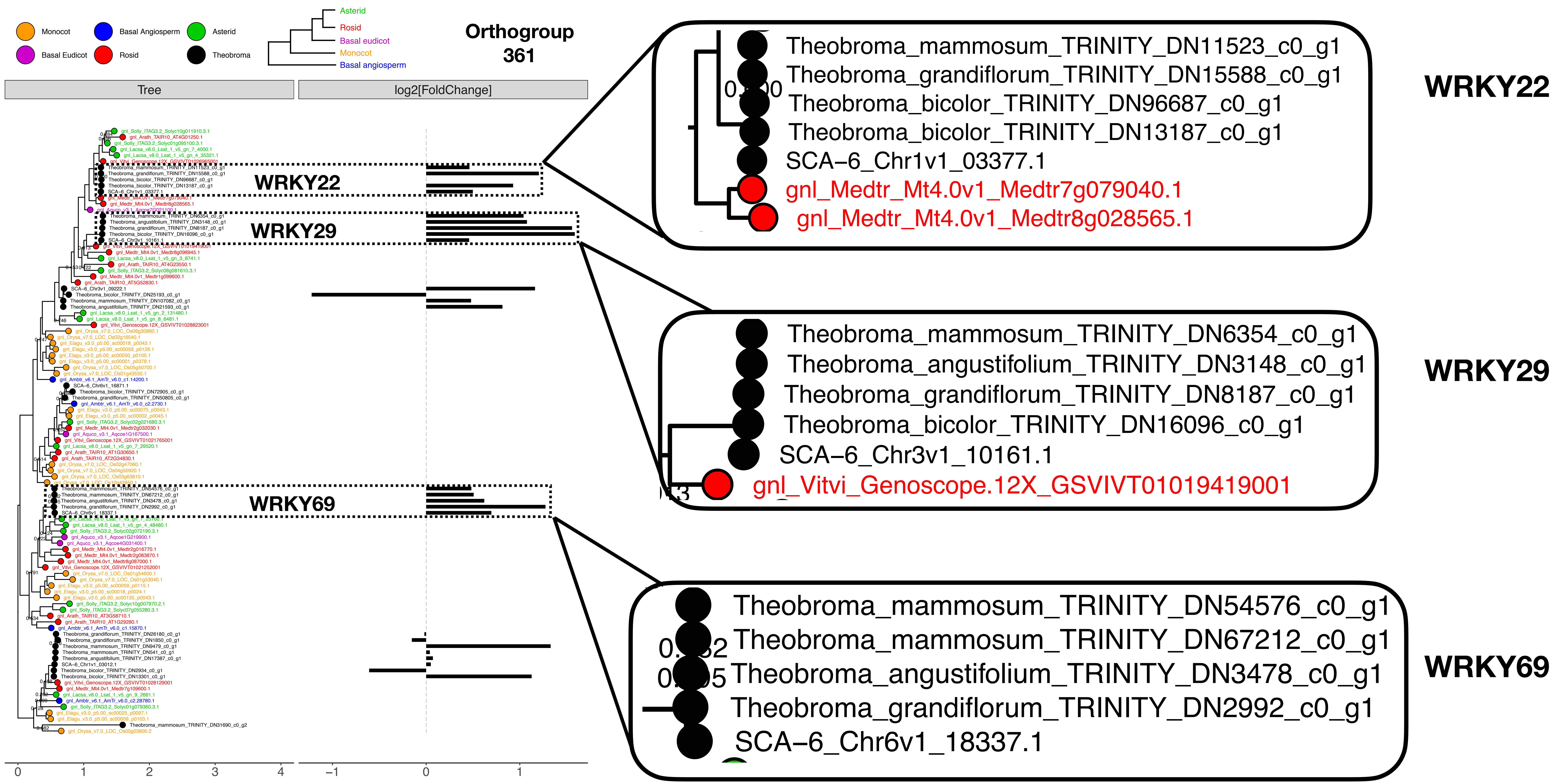

Supplemental Figure S12. Maximum-likelihood gene family phylogeny for orthogroup 361, WRKY transcription factors. Sequence IDs are colored according to their lineage: basal angiosperm (blue), basal eudicot (purple), monocot (orange), rosid (red), and asterid (green). All *Theobroma* species, including *T. cacao*, are shown in black. *T. cacao* sequences are from the SCA-6 genome. Node values indicate SH-like local supports calculated by FastTree v2. SH supports > 80 are not shown. Bars in the right panel indicate $\log_2$ fold changes. Boxes indicate the clades containing TcWRKY29 (SCA-6_Chr3v1_10161), TcWRKY22 (SCA-6_Chr1v1_03377), and TcWRKY69 (SCA-6_Chr6v1_18337), as well as their close orthologs across *Theobroma.* Codon alignments were created using MAFFT v7.205 (L-INS-i) and trees were inferred using FastTree v2.1.10 (-nt -gtr),

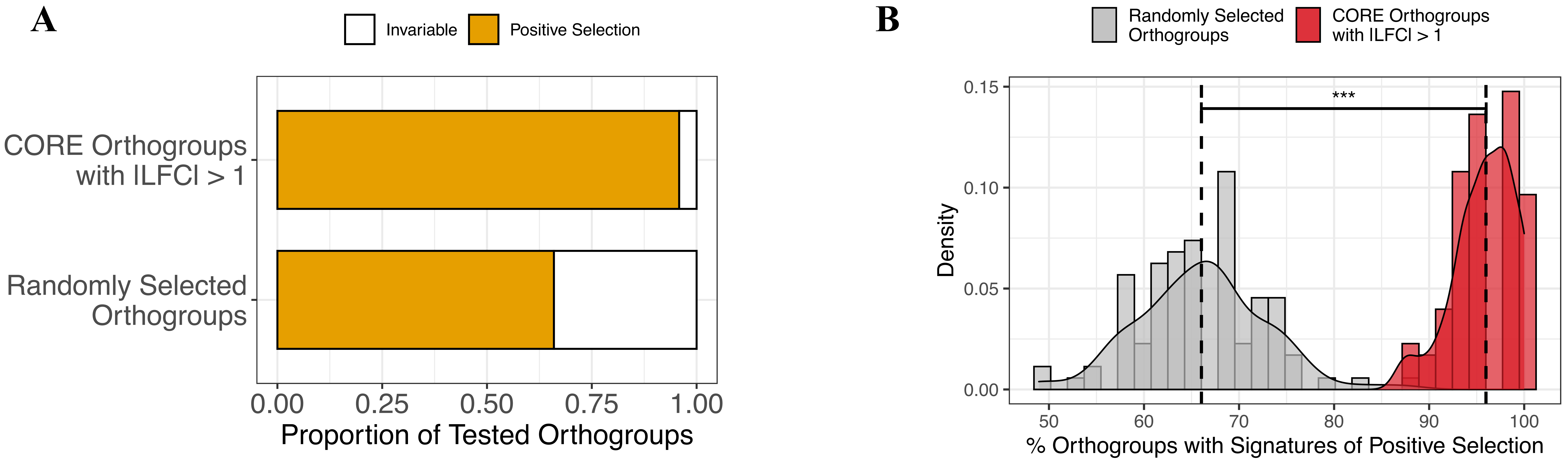

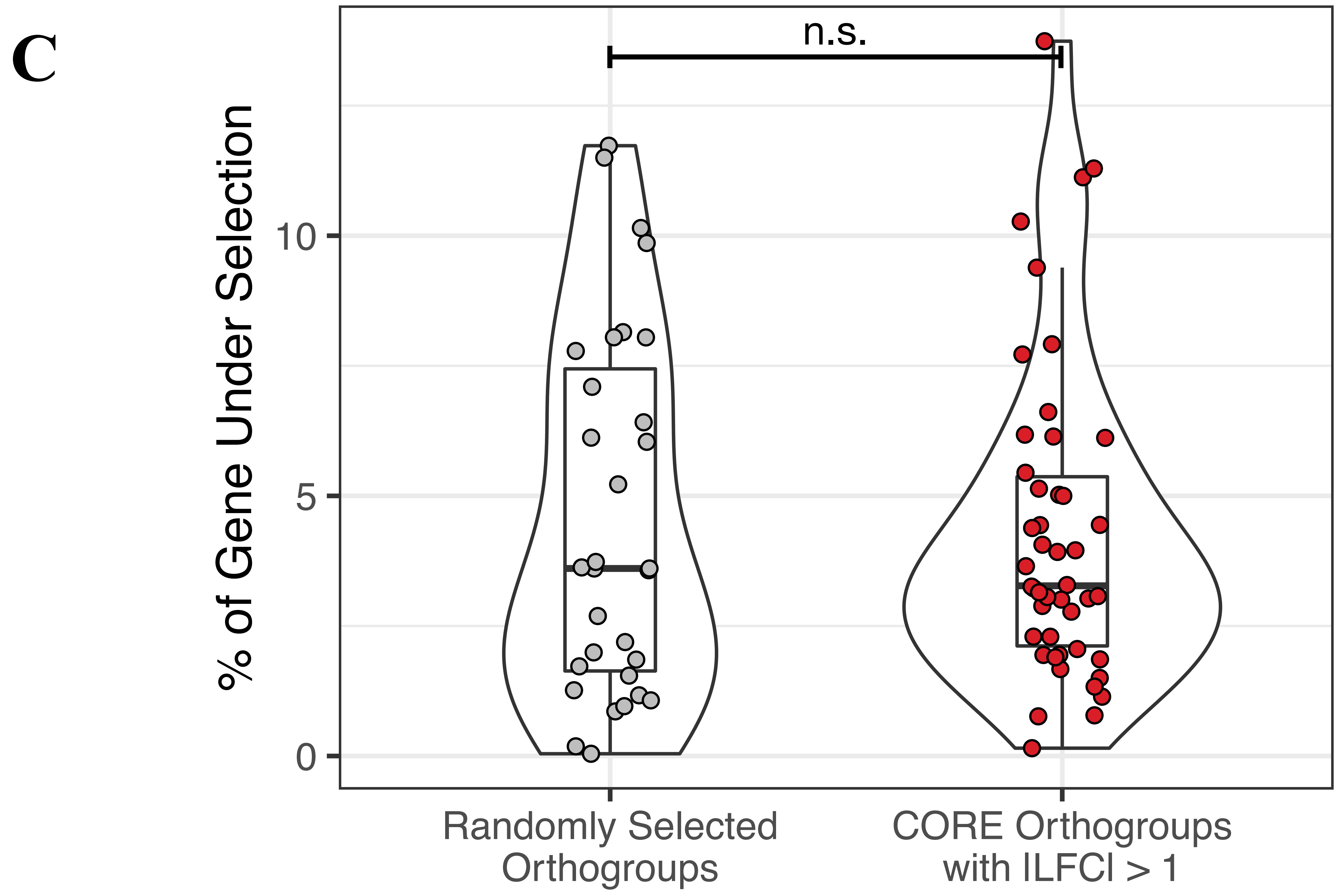

Supplemental Figure S13. Orthogroups with signatures of positive selection. (A) Proportion of orthogroups that have signatures of episodic, diversifying selection. CORE orthogroups with |LFC| > 1 in both *T. cacao* and non-cacao *Theobroma spp.* (top) were compared to an equal number (n = 48) of orthogroups drawn at random (bottom). Positive selection was significantly associated with orthogroup type (CORE vs random) (chi-sq. goodness-of-fit, $p < 0.001$). (B) Distribution of bootstrap replicates for both CORE orthogroups with |LFC| > 1 in both *T. cacao* and non-cacao *Theobroma spp.* (red), and orthogroups drawn at random (grey). The proportion of orthogroups displaying signatures of selection was significantly higher for CORE orthogroups with |LFC| > 1 in both *T. cacao* and non-cacao *Theobroma spp.* than for orthogroups drawn at random (t-test, $p < 0.001$). (C) The proportion of each gene under selection. CORE orthogroups with |LFC| > 1 in both *T. cacao* and non-cacao *Theobroma spp.* (red) were compared to an equal number of orthogroups drawn at random (grey). Differences were not significant (t-test, $p > 0.05$).
